# A Theoretical Perspective on the Actinic Photochemistry of 2-Hydroperoxypropanal

**DOI:** 10.1021/acs.jpca.2c03783

**Published:** 2022-07-28

**Authors:** Emanuele Marsili, Antonio Prlj, Basile F. E. Curchod

**Affiliations:** Centre for Computational Chemistry, School of Chemistry, University of Bristol, Bristol BS8 1TS, U.K.

## Abstract

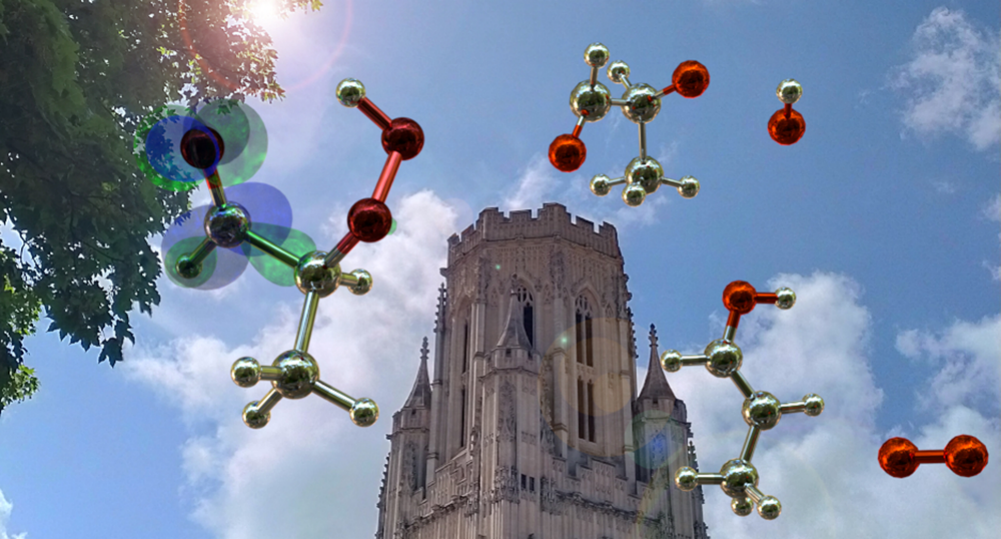

The photochemical reactions triggered by the sunlight absorption of transient volatile organic compounds in the troposphere are notoriously difficult to characterize experimentally due to the unstable and short-lived nature of these organic molecules. Some members of this family of compounds are likely to exhibit a rich photochemistry given the diversity of functional groups they can bear. Even more interesting is the photochemical fate of volatile organic compounds bearing more than one functional group that can absorb light—this is the case, for example, of α-hydroperoxycarbonyls, which are formed during the oxidation of isoprene. Experimental observables characterizing the photochemistry of these molecules like photoabsorption cross-sections or photolysis quantum yields are currently missing, and we propose here to leverage a recently developed computational protocol to predict in silico the photochemical fate of 2-hydroperoxypropanal (2-HPP) in the actinic region. We combine different levels of electronic structure methods—SCS-ADC(2) and XMS-CASPT2—with the nuclear ensemble approach and trajectory surface hopping to understand the mechanistic details of the possible nonradiative processes of 2-HPP. In particular, we predict the photoabsorption cross-section and the wavelength-dependent quantum yields for the observed photolytic pathways and combine them to determine in silico photolysis rate constants. The limitations of our protocol and possible future improvements are discussed.

## 1 Introduction

Atmospheric volatile organic compounds (VOCs) are potentially reactive molecules produced both anthropogenically and biogenically that can impact both atmospheric heat balance and air pollution. Isoprene, an exemplary and ubiquitous VOC, is emitted primarily from vegetation in quantities comparable to methane.^1^ The degradation of isoprene is mainly initiated by OH radicals, creating a complex network of oxidative reactions that generate multifunctional compounds with one or several oxygenated groups (e.g., hydroxides, hydroperoxides, carbonyls).^2^ The chemical reactivity of isoprene in the troposphere has been extensively studied both experimentally and theoretically, unravelling the entangled network of chemical reactions subsequently included in general atmospheric models such as the master chemical mechanism (MCM).^3−6^ The MCM model contains the empirical knowledge of products and kinetics of most relevant VOC reactions, allowing one to simulate the composition of the atmosphere in various environments (e.g., urban or tropical). The MCM serves as a near-explicit representation of the degradation mechanisms for VOCs, and it has been widely utilized to predict phenomena such as the formation of secondary organic aerosols (SOA)^7^ or the fluctuations in concentrations of tropospheric oxidant species.^8^ Nevertheless, the experimental data for a vast number of VOC reactions are not available, especially those involving highly unstable, transient VOC species that are difficult to study in laboratory conditions. This lack of experimental data often prompts the use of structure–activity relationships (SARs), assuming that the relevant properties can be estimated based on experimental data available for chemically similar compounds.

Despite the large number of reactions included in the MCM model, some of its predictions were inconsistent with real atmospheric measurements. An unexpectedly high OH radical concentration registered over the Amazon forest suggested that isoprene degradation recycles OH radicals much more efficiently than previously thought.^9^ This finding was linked with unexplored reaction pathways—occurring at low and moderate NO levels—in which hydroxy-isoprenyl-peroxy radicals undergo unimolecular isomerization reactions, today known as the Leuven isoprene mechanism (LIM).^10−12^ The key features of the LIM comprise (i) a direct recycling of OH via 1,5-H shift of the β-hydroxy-isoprenyl-peroxy radical^13^ and (ii) the formation of hydroperoxyaldehydes (HPALDs) via 1,6-H shifts of the isoprenyl-peroxy radicals,^14,15^ followed by a subsequent OH regeneration upon HPALDs photolysis.^16−18^ Importantly, the development of the LIM mechanism suggests that some photochemical reactions may be missing in the MCM—which is currently being largely based on ground-state reactivity.

α-hydroperoxycarbonyls are multifunctional VOCs structurally similar to HPALDs. They are formed through isoprene oxidation^19,20^ or as intermediates in the ozonolysis of ethylene.^21^ In analogy with HPALD, the photochemistry of α-hydroperoxycarbonyls deserves a thorough investigation, as these systems can serve as a potential OH recycling channel with important consequences on the oxidative balance in the troposphere.

Nevertheless, the photochemistry of multichromophoric VOC molecules like α-hydroperoxycarbonyls is tremendously challenging to investigate experimentally. Consequently, photolysis rate constants (*J*)—the first-order decay constants describing the kinetics of photolytic processes—are not directly available for most relevant α-hydroperoxycarbonyls. A recent work has experimentally estimated a sizable photolysis rate constant for 3-hydroperoxy-4-hydroxybutan-2-one, a molecule from the family of α-hydroperoxycarbonyls, hypothesizing that it would release OH upon light absorption.^22^ In addition, theoretical and computational photochemistry was used to unravel the photodissociation mechanism of simple α-hydroperoxycarbonyls, identifying a 1,5-H shift followed by the elimination of O_2_ as a primary photolytic pathway and estimating the corresponding *J* values.^23^ Photolysis via internal conversion was predicted to be faster than the processes involving intersystem crossing (ISC) based on SAR considerations.

**Figure 1:**
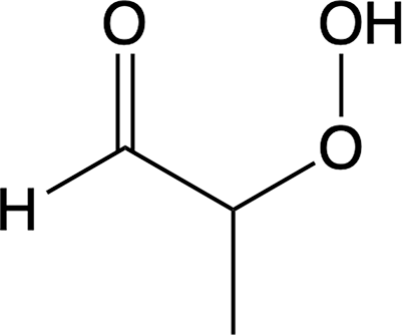
Chemical structure of 2-HPP.

In this work, we propose a different perspective to study the photochemistry of α-hydroperoxycarbonyls. Making use of recent developments in computational photochemistry and nonadiabatic excited-state molecular dynamics simulations, we build upon a recently proposed protocol^24^ to determine the two ingredients required to calculate *J*, namely, (i) the photoabsorption cross-section and (ii) the wavelength-dependent photolysis quantum yields. As an instructive model system, we examine 2-hydroperoxy-propanal (2-HPP, Figure 1)—one of the smallest molecules discussed in ref (23). The fully in silico protocol—based on quantum-chemical calculations and excited/ground-state dynamics simulations—gives us direct access to experimental observables connected to the photolysis of 2-HPP and offers insights into the mechanisms underlying each of the possible photochemical pathways, both in the excited and ground electronic state.

## 2 Methods

### 2.1 In Silico Photolysis Rate Constants

Following photoexcitation by sunlight, an atmospheric VOC can undergo different photochemical processes. Among them, the electronically excited VOC can suffer photolysis, leading to the formation of different products. A given photolysis process is characterized by its photolysis rate constant *J* —a first-order decay constant—defined as follows.
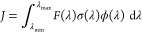
1The terms forming the integrand of eq 1 are defined as follows: *F*(λ) is the flux of the irradiation source (e.g., the solar actinic flux), σ(λ) is the photoabsorption cross-section of the molecule that undergoes photolysis, and ϕ(λ) is the wavelength-dependent quantum yield. Hence, the integrand of eq 1 says that, at a given wavelength λ, one has a flux of photon *F*(λ) with energy  coming from a given source that can possibly excite the molecule of interest with a cross-section σ(λ), and such electronic excitation can lead to the formation of a given photolysis product with a yield ϕ(λ) . By integrating this product within a spectral range of interest from λ_min_ and λ_max_ (e.g., the actinic region in approximate range from 280 to 360 nm), we can determine the overall photolysis rate constant.

Photolysis rate constants are required in chemical mechanisms to adequately account for possible direct photochemical processes induced by the sunlight absorption of VOCs. However, such a quantity can be challenging to determine experimentally for transient/unstable VOCs as 2-HPP. Interestingly though, some of the key ingredients forming the photolysis rate constant, namely, the photoabsorption cross-section σ(λ) and the quantum yield ϕ(λ), can nowadays be estimated using computational photochemistry, and we recently developed a computational protocol to determine the photolysis rate constants for a given VOC fully in silico.^24^ In the following, we briefly describe the key steps of this protocol.

The photoabsorption cross-section σ(λ) can be readily estimated by employing the nuclear ensemble approach (NEA).^25^ The NEA proposes to sample different nuclear geometries from a probability density constructed for the ground electronic and vibrational state of the molecule of interest and, then, to project each of these geometries onto the desired excited electronic states. By combining all the calculated transition energies and oscillator strengths, one can reconstruct a photoabsorption cross-section. A Wigner distribution for (uncoupled) harmonic oscillators is often employed as a probability density for the sampling, as it is particularly simple and convenient to construct—necessitating only an optimized ground-state geometry and corresponding vibrational frequencies. For atmospheric molecules that are not too flexible and for which a proper level of electronic structure theory can be selected, the NEA combined with Wigner sampling reproduces reasonably well the positions of the different bands forming a photoabsorption cross-section as well as their shape and corresponding intensity.^26−30^ Importantly, the NEA cannot reproduce vibronic progressions.

The wavelength-dependent quantum yield ϕ(λ) can be predicted by using nonadiabatic (i.e., excited-state) molecular dynamics simulations, which allow us to investigate the products formed after photoexcitation and their respective yields. For molecular systems in their full dimensionality, methods like the ab initio multiple spawning^31−34^ or trajectory surface hopping (TSH) would be preferred.^35^ TSH is a mixed quantum-classical approach that represents the excited-state dynamics of a molecule by a swarm of classical trajectories that can hop between electronic states as a result of non-Born–Oppenheimer effects, that is, when electronic states come close in energy and are coupled by the nuclear motion—the so-called nonadiabatic effects.^36^ The wavelength dependence of the quantum yield can be recovered by carefully selecting the initial conditions for the nonadiabatic dynamics. As stated in the previous paragraph, one can reproduce the photoabsorption cross-section σ(λ) by sampling geometries from a Wigner distribution. The Wigner distribution can also provide for each nuclear geometry selected a set of nuclear momenta, and the combination of a nuclear geometry plus nuclear momenta constitutes a given initial condition for the nonadiabatic dynamics. Hence, by dividing the calculated σ(λ) in different excitation windows, we can select for each window a set of initial conditions for the nonadiabatic dynamics. Once the excited-state dynamics for each of these initial conditions has been performed, one can monitor the formed photoproducts and assign, for each wavelength window, a ratio of photoproducts—a proxy for a quantum yield at this given wavelength. Combining all the windows will therefore provide an estimation of the wavelength-dependent quantum yield for each photolysis product.

A critical aspect for the success of all methods discussed above is an adequate choice of the electronic structure method. Linear-response time-dependent density functional theory (LR-TDDFT)^37^ or algebraic diagrammatic construction up to second-order (ADC(2)) and its spin component scaled (SCS) variant^38,39^ are among the simplest approaches compatible with excited-state dynamics.^40,41^ While they can provide an adequate description of electronic states around the Franck–Condon (FC) region,^42−45^ these methods have clear limitations when the ground and first excited state come close in energy, a situation very common in photochemistry. For instance, for carbonyl-containing molecules, ADC(2) may exhibit nonreactive conical intersection between S_1_ and S_0_ if the first excited state has an nπ* character. Our recent study showed that such intersections are an artifact of inadequate electronic structure description.^46^ For all these problematic cases, the use of a multiconfigurational method like state-averaged complete active space self-consistent field (SA-CASSCF)^47^ or multireference strategies like extended multistate complete active space second-order perturbation theory (XMS-CASPT2)^48,49^ is often required.

### 2.2 Computational Details

#### 2.2.1 Photoabsorption Cross-Section and Initial Conditions

The 12 conformers of 2-HPP identified in ref (23) were optimized with SCS-MP2 and a def2-SVP basis set.^50−52^ Harmonic vibrational frequencies at the ground-state minima and the thermochemistry were evaluated employing the same level of theory. On the basis of the calculated free energies, the seven conformers within 10 kJ/mol from the global minimum (conformer 1a—see Figure S1 in the Supporting Information for a depiction of the different conformers) were selected, and their photoabsorption cross-section was calculated using the NEA/Wigner approach.

For each conformer, a Wigner distribution for uncoupled harmonic oscillator was constructed and used to sample 500 geometries. For each geometry, vertical transitions and oscillator strengths were evaluated with SCS-ADC(2)/def2-SVP. All spectral transitions were broadened with Lorentzians using a phenomenological broadening of 0.05 eV. The resulting photoabsorption cross-section for each conformer was obtained by averaging the contribution of all 500 geometries using the NEA. The total photoabsorption cross-section for 2-HPP was then calculated by adding the contribution from each conformer, scaled by the appropriate Boltzmann factor. The NEA and the spectrum were calculated with Newton-X version 2.0.^53^

All SCS-MP2 and SCS-ADC(2)^39,43^ calculations reported in this work were performed with frozen core and the resolution of the identity (RI)^54^ using Turbomole 7.3.^55^ The D_1_ diagnostic was employed as an approximate measure of the multireference character of MP2 ground state.^56^

#### 2.2.2 Critical Points on Potential Energy Surfaces and Linear-Interpolation in Internal Coordinates

For the lowest-energy conformer showing an intramolecular hydrogen bond (1a), different critical points on the potential energy surfaces of 2-HPP were located. The FC point, that is, the ground-state minimum energy geometry, the S_1_ minimum, and a transition state toward a proton-coupled electron transfer in S_1_ were obtained with SCS-ADC(2)/def2-SVP. Minimum-energy conical intersections (MECIs), the biradical ground-state minimum and transition state were located with XMS(3)-CASPT2(12/9) with a cc-pVDZ^57^ basis set using BAGEL 1.2.^58^ All XMS-CASPT2 calculations reported in this work used density fitting, the SS-SR contraction scheme, and a real vertical shift of 0.5 hartree. Linear interpolation in internal coordinates (LIIC) pathways were generated to connect the different critical points located. The active space for XMS-CASPT2 calculations was designed to ensure a proper description of the potential energy surfaces for the proton-coupled electron transfer, the ^1^O_2_ release, and the OH and OOH photodissociation as well as the conservation of total energy during the excited-state and ground-state dynamics. The orbitals forming the active space and their evolution along the proton-coupled electron transfer pathway are presented in the Supporting Information (Figures S2 and S3). The orbitals included are *n*′(OO), σ(CO), σ(OO), π(CO), two combinations of *n*′(OO) and *n*(CO), π*(CO), σ*(OO), and σ*(CO).

#### 2.2.3 Excited-State Dynamics and Quantum Yields

The excited-state (nonadiabatic) dynamics simulations were performed with the (fewest-switches) TSH algorithm.^36^ The nonadiabatic couplings were obtained by using the wave-function overlap scheme, and the kinetic energy was adjusted by rescaling the nuclear velocity vector isotropically following a successful hop. The electronic populations were corrected to prevent overcoherence using the energy-based decoherence correction of Granucci and Persico.^59^

All TSH trajectories were initiated in the first excited electronic state S_1_, starting from initial conditions sampled randomly from a harmonic Wigner distribution for conformer 1a (and 1c) (see Table S1). In total, 166 (conformer 1a) and 80 (conformer 1c) trajectories were propagated. Because of the cost of the TSH calculations, we only selected the conformers 1a and 1c, as they are the two lowest-energy conformers with and without an intramolecular hydrogen bond (based on the CCSD(T)-F12/cc-pVDZ-F12//M06-2X-D3/6-311++G(2d,p) free-energy calculations in ref (23)).

All TSH dynamics were performed using SCS-ADC(2)/def2-SVP for the electronic structure, with a time step of 0.5 fs using Newton-X coupled with Turbomole.^40^ The TSH trajectories were propagated for a maximum of 100 ps. All trajectories exhibiting an artificial nonreactive conical intersection^46^ (NRCI) with S_0_ were discarded (their statistics is discussed in Section 3.3).

A special treatment was required for those trajectories approaching the S_1_/S_0_ intersection seam following a proton-coupled electron transfer in S_1_ due to the enhanced biradical character of the ground electronic state and the need for an adequate description of the intersection seam and nonadiabatic transitions (see Section 3.1 for a full discussion). In this particular case, a TSH trajectory using SCS-ADC(2)/def2-SVP would be terminated when the S_1_/S_0_ energy gap gets lower than 0.01 eV. One would then backtrack the trajectory up until 15 fs, monitoring the value of the D_1_(MP2) diagnostic to determine the last time along the SCS-ADC(2) TSH trajectory that can be trusted, that is, when the D_1_ diagnostic is less than 0.075 following the recommendation of ref (56) (note that large D_1_ values indicate increased multireference character of the ground state). The nuclear coordinates and velocities at this specific time then serve as a restart for the TSH dynamics now employing XMS(3)-CASPT2(12/9)/cc-pVDZ, using a time step of 0.25 fs (the reduced time step is necessary due to the high kinetic energy of the trajectories when transferring back to S_0_). A check is performed to make sure the correct set of orbitals (shown in Figure S2) is recovered at the restarting geometry. For the rare cases where the adequate set of orbitals (and electronic-state characters) could not be retrieved, we move to the closest time step for which the proper orbitals could be obtained. Figure S7 shows the excellent agreement between the electronic energies along a TSH/SCS-ADC(2) trajectory and those recalculated with XMS(3)-CASPT2(12/9), even in cases of diabatic trappings. The validity of this switch between methods is further discussed in Section 3.1. In total, four trajectories (one for 1a, three for 1c) have been discarded (see Table S1), as the proper active space for XMS(3)-CASPT2(12/9) could not be obtained for geometries within the 15 fs of dynamics preceding the crossing point.

For the trajectories leading to the ^1^O_2_ release, the strong destabilization of the closed-shell character can lead to instabilities in the XMS(3)-CASPT2(12/9) calculations. We closely monitored the variation of the total energy along these trajectories: for each trajectory, if more than four discontinuities of more than 0.05 eV each in the total energy were observed (but less than 0.25 eV—only three trajectories for 1a and none for 1c exhibited a discontinuity of more than 0.2 eV), the trajectory was stopped and restarted before the first discontinuity occurred, using XMS(2)-CASPT2(12/9). In XMS(2)-CASPT2(12/9), only the two lowest electronic states with a biradical character are taken into account. The validity of this approach has been confirmed by comparing the results of XMS(3)-CASPT2(12/9) and XMS(2)-CASPT2(12/9) along a trajectory (see Figure S8 in the Supporting Information)—the third electronic state splits from the two electronic states with a biradical character. All TSH dynamics employing XMS-CASPT2 were performed with the SHARC 2.1 code.^60,61^

#### 2.2.4 Spin–Orbit Coupling Matrix Elements

Spin–Orbit coupling matrix elements were calculated with SA(3S,3T)-CASSCF(12/9)/cc-pVDZ using Molpro 2012.1.,^62,63^ using the same active space as for all XMS-CASPT2 calculations. For each matrix element calculated, we ensured that the electronic character of the states considered matched between SA-CASSCF and SCS-ADC(2). The magnitude of the spin–orbit coupling between the singlet S_1_ and triplet T_*n*_ electronic state is calculated as , where *M*_S_ = −1, 0, 1 are the triplet sublevels of T_*n*_ and  the (complex) spin–orbit coupling matrix element between the singlet electronic state S_1_ and the spin sublevel *M*_S_ of the triplet electronic state T_*n*_.

## 3 Results and Discussion

In this Section, we first highlight the possible photochemical pathways that 2-HPP can follow after photoexcitation (Section 3.1) and use these different pathways as a way to benchmark the levels of theory and computational strategies that will be employed for the excited-state dynamics simulations. We then focus on the simulation of the photoabsorption cross-section σ(λ) of 2-HPP (Section 3.2), comparing our theoretical results to earlier work employing SARs. Using the calculated photoabsorption cross-section, we performed excited-state dynamics simulations at different excitation wavelengths, resulting in the generation of theoretical wavelength-dependent quantum yields (ϕ(λ)) for the formed photoproducts and the identification of interesting dynamical processes in the excited electronic states (Section 3.3). In Section 3.4, we discuss the importance of intersystem crossing processes in light of our simulations.

### 3.1 Potential Photochemical Pathways

We focus here on the main reaction channels of 2-HPP when photoexcited to S_1_ and without intersystem crossing, namely, a proton-coupled electron transfer followed by ^1^O_2_ release, OH photodissociation, and OOH photodissociation. This Section will present not only pathways and scans characterizing these processes but also a stringent benchmark of the level of electronic structure theory prior to any excited-state dynamics. In the particular case of 2-HPP, we will compare SCS-MP2/ADC(2) and XMS-CASPT2—the two methods that will be used throughout this work.

#### 3.1.1 Excited-State Proton-Coupled Electron Transfer and ^1^O_2_ Release

**Figure 2:**
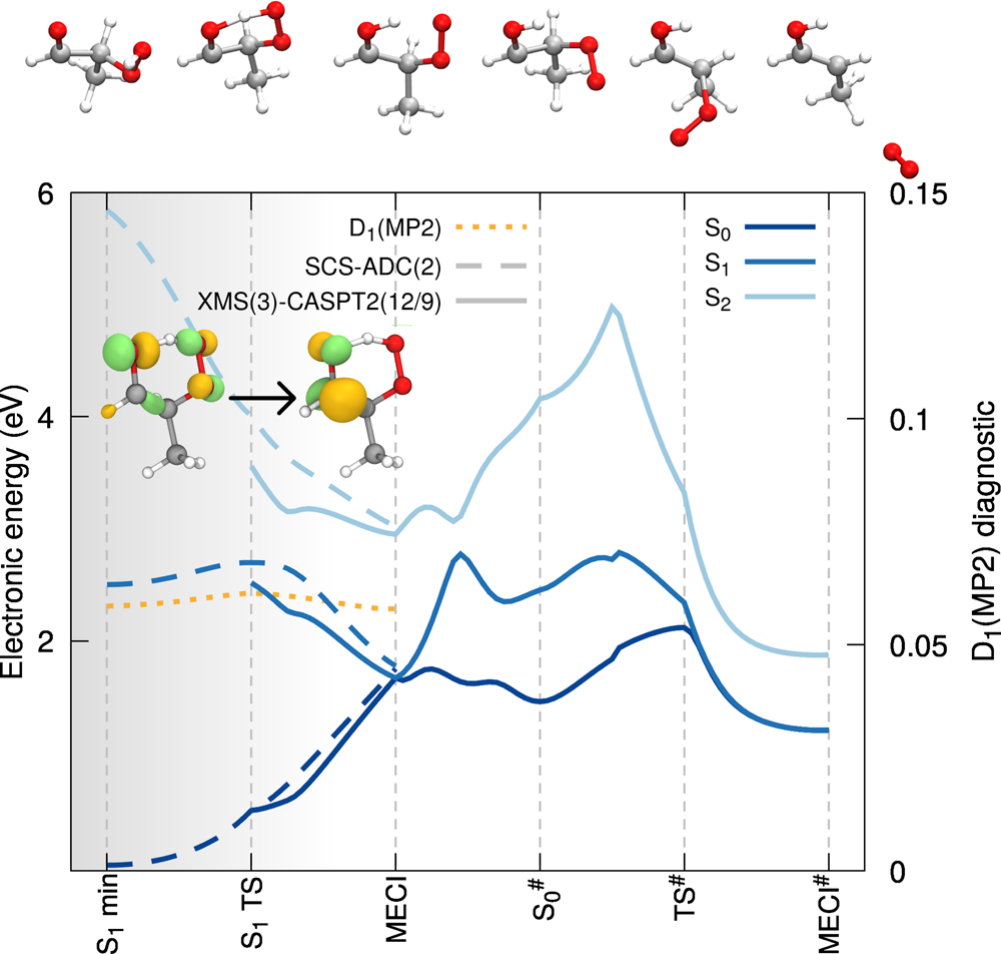
LIIC pathways for the excited-state proton-coupled electron transfer and ^1^O_2_ release. Comparison of the electronic energies obtained with SCS-ADC(2)/def2-SVP (dashed lines) and XMS(3)-CASPT2(12/9)/cc-pVDZ (solid lines) for the three lowest electronic states, S_0_ (dark blue), S_1_ (blue), and S_2_ (light blue). The S_1_ minimum and S_1_ TS geometries were obtained at the SCS-ADC(2)/def2-SVP level of theory, while the critical geometries for the S_0_/S_1_ MECI, biradical S_0_ minimum (S_0_^#^), biradical S_0_ TS^#^, and O_2_ dissociation MECI (MECI^#^) were optimized with XMS(3)-CASPT2(12/9)/cc-pVDZ. The inset shows natural transition orbitals (NTOs) characterizing the electronic character of S_1_ at the S_1_ TS geometry. The upper panel shows the molecular structure corresponding to each critical point located. The D_1_ diagnostic along the pathway for the SCS-MP2 ground state is given with a dotted orange line. The shaded area highlights the region of the LIIC where SCS-MP2/ADC(2) could be trusted.

First we analyze the possible photochemical pathways of 2-HPP with an excited-state proton-coupled electron transfer. This process, taking place exclusively on the first excited singlet state S_1_, is related to the mechanism described in ref (23) as 1,5-H shift. Our choice of nomenclature will be explained soon. The characterization of this process begins by localizing the minimum (S_1_ min, having a *n*(O) →π*(CO) character) and transition state (S_1_ TS) in S_1_ using SCS-ADC(2)/def2-SVP (Figure 2). Interestingly, the electronic character of the S_1_ state at the S_1_ TS structure (inset of Figure 2) corresponds to a (*n*(O) + *n*′(OO)) → π*(CO) transition, highlighting that the transfer of a proton from the hydroperoxide to the carbonyl takes place with the simultaneous displacement of the part of electron density in the same direction. This observation prompts us to call this process an excited-state proton-coupled electron transfer, as an H atom transfer would imply that the electron would be localized on the proton during the transfer. A proton following its path toward the carbonyl group implies the location of an MECI between S_1_ and the ground state S_0_. This critical geometry was localized with XMS(3)-CASPT2(12/9)/cc-pVDZ due to its inherent multireference character.

The electronic energies at the critical geometries discussed up to now can be connected by performing a linear interpolation between the structures using internal coordinates and calculating electronic energies for each intermediate geometry. Such LIIC pathways highlights important features of the potential energy surfaces between critical points but should not be confused with minimum-energy pathways. LIICs are represented by lines in Figure 2—dashed lines are electronic energies calculated with SCS-MP2/ADC(2)/def2-SVP and plain lines with XMS(3)-CASPT2(12/9)/cc-pVDZ. We note that XMS(3)-CASPT2(12/9) suffers from orbital rotations and is unstable between the FC and S_1_ min critical geometries due to a higher density of high-lying electronic states in this region of configuration space. The electronic energy difference between S_1_ TS and the S_1_ min is 0.20 eV, while the FC point lies 0.72 eV above the S_1_ min (SCS-ADC(2)/def2-SVP). We note that the LIIC between the FC point and the S_1_ min is given in the Supporting Information (Figure S5). The LIIC pathways highlight the sharp decrease of S_1_ energy from the S_1_ to the MECI. Importantly, SCS-ADC(2)/MP2 appears to reproduce qualitatively well the shape of the XMS-CASPT2 potential energy curves when approaching the MECI.

From the MECI point, the molecule can funnel back toward the S_0_ minimum of the original molecule with a back-transfer of the proton to the peroxide. Proceeding toward the release of ^1^O_2_ after passage through the MECI, though, requires a biradical, multiconfigurational character of the molecule in the ground electronic state. The corresponding ground-state minimum (S_0_^#^) was optimized with XMS(3)-CASPT2(12/9)/cc-pVDZ and preserves the proton on the original carbonyl group (see the upper panel of Figure 2). Focusing on the LIIC pathways between MECI and S_0_^#^, one can observe a steep rise in the S_1_ electronic energy, which, from the MECI point, picked up a closed-shell character that is highly destabilized. The closed-shell character later even transferred to S_2_ (avoided crossing midway between MECI and S_0_^#^). The release of ^1^O_2_ requires the passage through a transition state in the biradical ground state (TS^#^), which eventually leads to an MECI with S_1_ (MECI^#^), where the dissociation takes place. At the TS^#^ structure, the carbon bearing the original hydroperoxide group displays a significant sp^2^ character (see the structure in the upper panel of Figure 2), while its bond with the OO group is strongly elongated (1.78 Å).

As attested by Figure 2, the release of ^1^O_2_ implies a rather complex series of events on the S_1_ and S_0_ potential energy surfaces. From an electronic structure perspective, the appearance of a strong biradical character in the ground electronic state following the proton-coupled electron transfer hampers the use of SCS-MP2/ADC(2) from just before the region of the first MECI. SCS-MP2/ADC(2) can, however, be safely used from the FC region until just after the S_1_ TS is passed, as confirmed by a comparison with XMS-CASPT2 presented in the Supporting Information (Figure S7). From a dynamics perspective, reaching the S_1_ TS can take several picoseconds, a time scale that prohibits the use of XMS(3)-CASPT2(12/9) dues to its high computational cost, in contrast to the computationally affordable SCS-ADC(2). Hence, we are forced to adopt a combination of two methods. The excited-state dynamics of 2-HPP will be conducted with SCS-ADC(2) up until it approaches the S_1_/S_0_ intersection seam following a proton-coupled electron transfer. We then rewind the trajectory by up to 15 fs and restart the S_1_ dynamics with XMS(3)-CASPT2(12/9) from there—making sure that the restart point is far enough from the S_1_/S_0_ intersection in terms of energy and that the correct active space is recovered. The close agreement between SCS-ADC(2) and XMS-CASPT2 discussed above before the MECI region comforts us in this switch of methods. The dynamics can proceed and describe adequately the nonadiabatic transitions between S_1_ and S_0_ and the subsequent S_0_ and S_1_ dynamics with a biradical reference up until the O_2_ dissociation. We note that the return to the FC region of 2-HPP can also be described by this strategy. While not being perfectly satisfactory, the blended approach proposed here is a compromise to simulate the entire nonradiative decay of 2-HPP leading to the formation of ^1^O_2_ (the shaded area in Figure 2 highlights when SCS-MP2/ADC(2) can be trusted).

#### 3.1.2 OH and OOH Photodissociation

**Figure 3:**
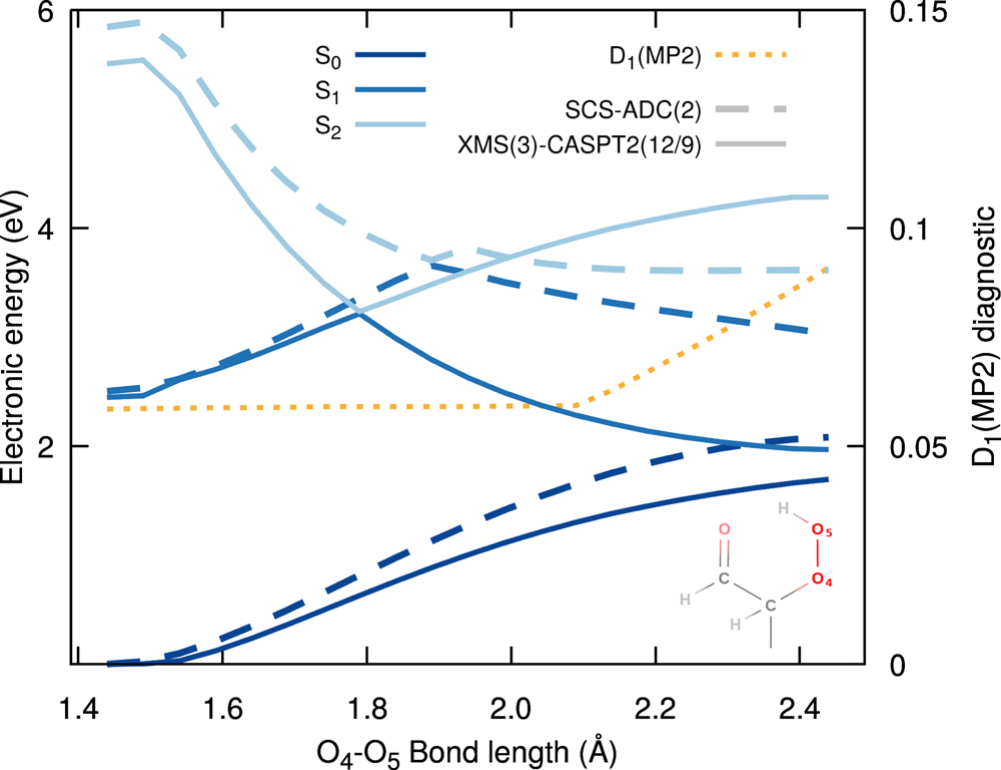
OH release in the excited state. Rigid scan along the O_4_–O_5_ bond of 2-HPP (see inset) starting from the S_1_ min geometry obtained at SCS-ADC(2)/def2-SVP level of theory. Comparison of the electronic energies obtained with SCS-ADC(2)/def2-SVP (dashed lines) and XMS(3)-CASPT2(12/9)/cc-pVDZ (solid lines) for the three lowest electronic states, S_0_ (dark blue), S_1_ (blue), and S_2_ (light blue). The D_1_ diagnostic along the scan for the SCS-MP2 ground state is given with a dotted orange line.

The photodissociation of OH in the first excited electronic state can be envisioned if a change of electronic character for S_1_ can occur, moving from an *n*(O) →π*(CO) character in the FC and S_1_ min region to an *n*′(OO) →σ*(OO) dissociative character. To investigate the interplay between the electronic states in such a process, one can start from the S_1_ min geometry and stretch the O–O of the hydroperoxide group without relaxing the molecular geometry. The electronic energies obtained from this rigid scan are presented in Figure 3. The S_1_ state is clearly destabilized at the beginning of the stretch as a result of its *n*(O) →π*(CO) character, in stark contrast with S_2_, which exhibits an *n*′(OO) →σ*(OO) nature. At the XMS-CASPT2 level of theory (solid lines in Figure 3), the change of character for the S_1_ state occurs at a O–O bond length just under 1.8 Å. After this point, the S_1_ electronic state gains an *n*′(OO) →σ*(OO) character and would lead to the photodissociation of a OH radical. While this process may appear simple enough to proceed efficiently, earlier dynamical studies on a C6-HPALD revealed that the very weak diabatic coupling between the two electronic-state characters protects the molecule from dissociation by forcing it to follow the same electronic character, that is, by a nonadiabatic transition to S_2_.^64^ Only a certain approach of the intersection seam may allow for the release of OH radicals. More information on this process, coined diabatic trapping, is provided in Section 3.3. The description of the OH photodissociation pathway provided by SCS-ADC(2) (dashed lines in Figure 3) presents the same qualitative features as with XMS-CASPT2. A switch of electronic character is observed, but at a bond length closer to 1.9 Å. The electronic-character exchange also takes place at a higher S_1_ electronic energy from the S_1_ min along this rigid scan in comparison to XMS-CASPT2. The D_1_(MP2) diagnostic increases sharply when the O–O bond is stretched beyond 2.0 Å, revealing an emerging multiconfigurational nature of the ground electronic state. In addition, the photodissociation of OH from a hydroperoxide group leads to the appearance of low-lying electronic states with a dominant doubly excited character—a type of electronic state that ADC(2) only approximates to zeroth order.^24^ In summary, SCS-ADC(2) is likely able to describe the switching between *n*(O) → π*(CO) and *n*′(OO) → σ*(OO) characters along S_1_ fairly well, but, as expected, this method does not properly describe the OH dissociation limit.

**Figure 4:**
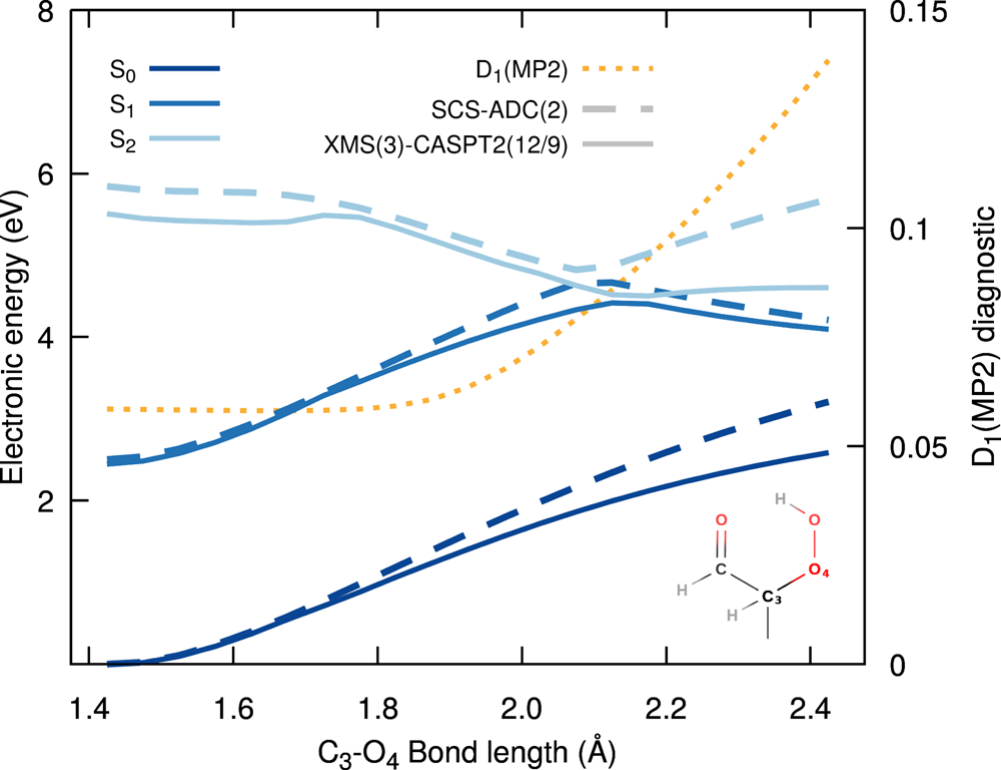
OOH release in the excited state. Rigid scan along the C_3_–O_4_ bond of 2-HPP (see inset) starting from the S_1_ min geometry obtained at SCS-ADC(2)/def2-SVP level of theory. Comparison of the electronic energies obtained with SCS-ADC(2)/def2-SVP (dashed lines) and XMS(3)-CASPT2(12/9)/cc-pVDZ (solid lines) for the three lowest electronic states, S_0_ (dark blue), S_1_ (blue), and S_2_ (light blue). The D_1_ diagnostic along the scan for the SCS-MP2 ground state is given with a dotted orange line.

The OOH release from S_1_ can be investigated by using a similar strategy, namely, a rigid scan from the S_1_ min structure along the C–O bond of the hydroperoxide group (Figure 4). The electronic energies obtained with XMS-CASPT2 and SCS-ADC(2) along this scan are in qualitative agreement, showing a crossing between S_1_ and S_2_ at just under 2.10 Å for SCS-ADC(2) and around 2.15 Å for XMS-CASPT2. The D_1_ diagnostic however increases dramatically after 2.0 Å. While care has to be taken in the analysis of rigid scans, the two methods appear to indicate that the OOH dissociation would be a far more energetically demanding process than the OH release.

### 3.2 Photoabsorption Cross-Section of 2-HPP

**Figure 5:**
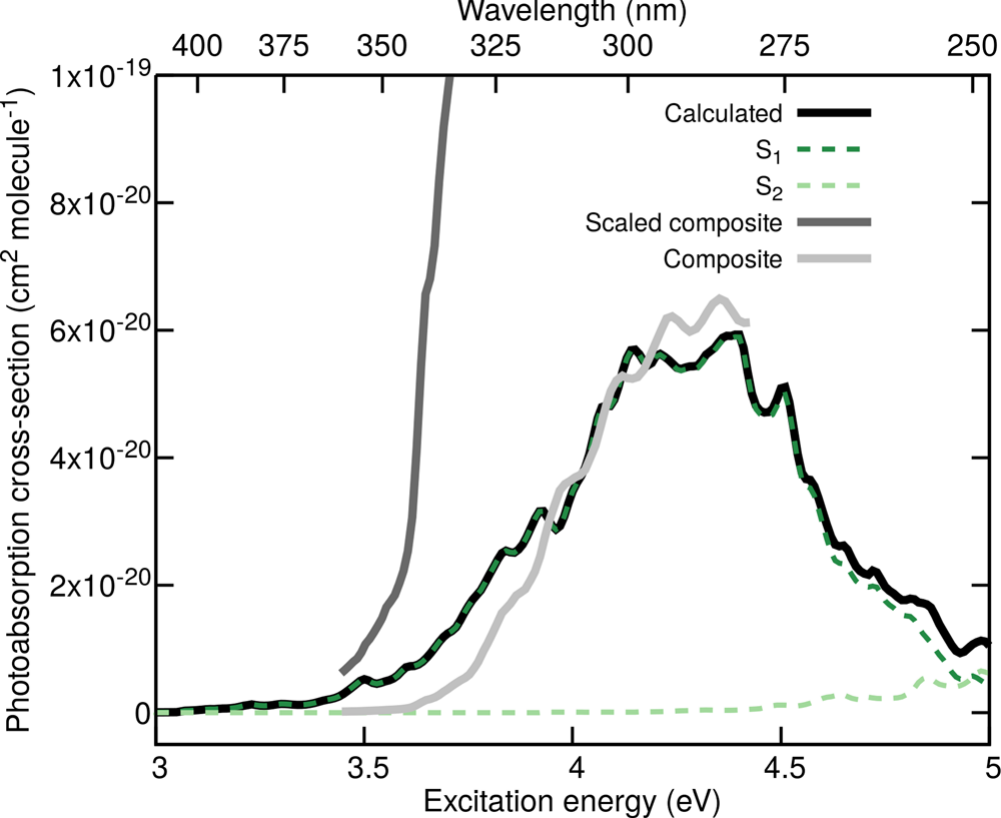
Calculated and SARs photoabsorption cross-sections of 2-HPP. The calculated photoabsorption cross-section obtained at the SCS-ADC(2)/def2-SVP level of theory is shown with a black solid line together with the S_0_ → S_1_ (dark-green dashed line) and S_0_ → S_2_ (light-green dashed line) contributions. The SARs composite photoabsorption cross-section of 2-HPP obtained by combining the experimental cross-sections of methylhydroperoxide and propanal (see ref (23) for details) is indicated with a light-gray solid line, while its scaled version is given with a dark-gray solid line. Both cross-sections are reproduced from ref (23).

The photoabsorption cross-section σ(λ) is one of the components required to characterize photolytic processes, as discussed in Section 2.1. We calculated the photoabsorption cross-section for the lowest conformers of 2-HPP (see Figure S1 for a depiction of the conformers) employing the NEA with the optimized S_0_ geometry and corresponding vibrational frequencies obtained with the SCS-MP2/def2-SVP and transition energies and oscillator strengths calculated with SCS-ADC(2)/def2-SVP (see Computational Details, Section 2.2, for additional information). The total photoabsorption cross-section (black line in Figure 5) was generated by adding together the individual cross-sections for each conformer, weighted by the appropriate Boltzmann factor to represent their theoretical population. The observed band in the actinic region stretches from 3.5 up to 5 eV with a center at 4.25 eV and is characterized by a very small cross-section. Decomposing the total cross-section into its electronic transitions (dashed lines in Figure 5) highlights the dominant contribution of the S_0_ → S_1_ excitation to the main band in the actinic region. This transition exhibits an *n*(O) → π*(CO) character located on the carbonyl moiety of 2-HPP, as expected from the minute cross-section in this region. Interestingly, the S_0_ → S_2_ contribution to the total cross-section (light-green dashed line in Figure 5), which exhibits an *n*′(OO) → σ*(OO) character and is therefore prone to trigger direct OH photodissociation, only appears to contribute for photon energies higher than 4.5 eV.

The experimental photoabsorption cross-section of 2-HPP is unfortunately unknown. Nevertheless, the theoretical photoabsorption cross-section obtained at the SCS-ADC(2) level of theory can be compared to earlier predictions based on SARs.^23^ 2-HPP is composed of a hydroperoxide moiety and an aldehyde functional group. Hence, earlier work has proposed to predict its photoabsorption cross-section by combining the experimental photoabsorption cross-section of methylhydroperoxide and propanal.^23^ The resulting composite cross-section is given as a light-gray solid line in Figure 5 and matches the theoretical prediction both from the location of the maximum of the low-energy band and its absolute cross-section. One possible shortcoming of the decomposition of 2-HPP in its functional group is the absence of possible intramolecular interaction—we have seen earlier that two of the low-energy conformers of 2-HPP possess an intramolecular H-bond between the hydroperoxide and carbonyl groups, possibly altering the *n*(O) →π*(CO) transition characterizing the low-energy band. A scaling factor was proposed to account for this possible intramolecular interaction, leading to the scaled composite cross-section displayed in dark gray in Figure 5. This scaled composite spectrum reaches much higher cross-section values, in less good agreement with our theoretical prediction. We note that the level of theory may also influence the overall intensity of the first band, as discussed recently for the theoretical determination of a photoabsorption cross-section for 2-HPP within DFT and LR-TDDFT.^30^ Both levels of theory appear to indicate that the 2-HPP photoabsorption cross-section may be slightly altered by intramolecular effects but, perhaps, not as much as suggested by the scaling factor employed in the SARs cross-section.

Overall, the photoabsorption cross-section of 2-HPP calculated indicate that, in the actinic region, 2-HPP is most likely to be photoexcited to the first electronic excited state S_1_ with an *n*(O) →π*(CO) character. On the basis of this result, the next step of our study consists in studying the possible photoproducts formed by photoexciting 2-HPP at different wavelengths within the low-energy band of the calculated photoabsorption cross-section.

### 3.3 Wavelength-Dependent Quantum Yield of 2-HPP and Formation of Photoproducts

In this Section, we focus on the formation of photoproducts following the photoexcitation of 2-HPP in the actinic region and the determination of wavelength-dependent quantum yields ϕ(λ)—another crucial component for photolysis rate constant as discussed in Section 2.1. Our analysis here is based on the conformer 1a, which exhibits an intramolecular hydrogen bond. We also investigated the conformer 1c, which does not have an intramolecular H-bond (the results are presented in the Supporting Information). We further note that fast interconversion between energetically close conformers takes place in the excited electronic states (see Figure S4 in the Supporting Information), further justifying the focus of this work on 1a, which naturally visits other minima following photoexcitation.

**Figure 6:**
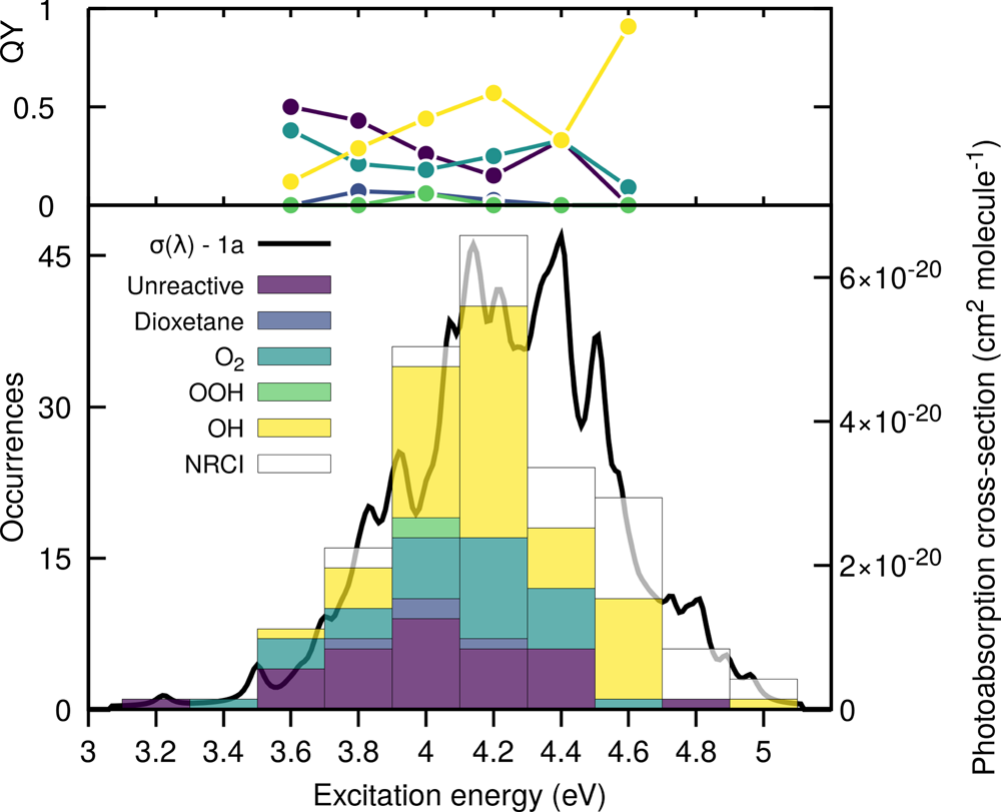
Wavelength-dependent photoproducts (lower panel) and quantum yields (upper panel) of 2-HPP. The photoproducts—OH and OOH dissociation, ^1^O_2_ release and formation of prop-1-en-1-ol, dioxetane formation, unreactive trajectories, NRCI—were obtained by simulating the excited-state and subsequent athermal ground-state dynamics of 2-HPP (conformer 1a) with TSH/SCS-ADC(2)/def2-SVP and TSH/XMS(3)-CASPT2(12/9)/cc-pVDZ. The occurrences (unweighted number of TSH trajectories ending as one of the photoproducts defined) of each reactive pathway are overlaid with the calculated photoabsorption spectra (SCS-ADC(2)/def2-SVP) for S_1_ state of the conformer 1a. The trajectories leading to an NRCI pathway were discarded from the quantum yield calculation (see Table S1 in the Supporting Information). The wavelength-dependent quantum yields were calculated only for windows with more than eight successful trajectories.

We emulated the wavelength-dependent photoexcitation of 2-HPP by defining a series of energy windows in the photoabsorption cross-section of 2-HPP (lower panel of Figure 6). We sampled a number of initial conditions randomly and grouped them by energy window; that is, we matched their transition energy to S_1_ to a given energy window. Each initial condition in each energy window was then used to initialize a TSH trajectory, using SCS-ADC(2) and XMS-CASPT2 (see Computational Details). Importantly, the TSH dynamics was not stopped as soon as a trajectory reached the ground electronic state S_0_ but was continued in S_0_ to account for possible athermal effects coming from the nonstatistical distribution of the internal energy gained by the photoexcitation.^65^ We start our discussion by showing the distribution of the photoproducts observed as a function of the photoexcitation energy, and we comment on the mechanisms leading to their formation in Section 3.3.1.

The TSH dynamics led to the observation of expected deactivation pathways based on our discussion in Section 3.1 and ref (23), like the dissociation of OH and OOH or the ^1^O_2_ release (Figure 6). The release of ^1^O_2_ with formation of prop-1-en-1-ol is one of the main pathways to the photoreactivity of 2-HPP at low excitation energies and occurs for ∼45% of all the trajectories undergoing a proton-coupled electron transfer. A significant number of unreactive trajectories were observed; this designation qualifies TSH trajectories that proceeded through a proton-coupled electron transfer mechanism but return to the (FC) region following the nonradiative pathway to S_0_. The OH photodissociation is already present in the low excitation energy windows but gains importance when exciting 2-HPP with higher-energy photons. It is important to note that all dynamics were initiated in S_1_ (see Figure 5), meaning that the variation in photoproducts is not caused by the excitation to a different electronic state (with a different electronic character) but by the opening of new reaction channels at higher excitation energies (see also Section 3.3.1). The photodissociation of OH from 2-HPP in its S_1_ excited state is a prime example of such a photochemical process involving a change in electronic character within a given electronic state, and its mechanism will be presented in Section 3.3.1. The photodissociation channel of OOH appears to be a minute contributor to the photochemistry of 2-HPP at all excitation energies sampled. Two trajectories remained in S_1_ for 100 ps without suffering any deactivation pathways. These trajectories are particularly interesting, as they could potentially be subjected to processes that are not described explicitly in our simulations, like intersystem crossings or collision with other molecules.^64^ Intersystem crossings from S_1_ to low-lying triplet states were proposed in ref (23) and will be discussed separately in Section 3.4 below. Our TSH dynamics also highlighted an unexpected (minor) reaction channel involving the formation of a dioxetane moiety following a proton-coupled electron transfer event (see Section 3.3.1 for the mechanistic details). We finally note that the use of SCS-ADC(2) for our TSH dynamics of 2-HPP implies that some artificial NRCIs might be visited during the dynamics (as discussed in the Computational Details). Such processes are given by white boxes in the lower panel of Figure 5 for transparency but not included in the determination of the wavelength-dependent quantum yields (upper panel of same figure). As such, the only impact of these events on our simulations is the loss of trajectories contributing to the statistics of the quantum yields.

In summary, the TSH trajectories appear to indicate that the 2-HPP photochemistry in the actinic region is dominated by a proton-coupled electron transfer process that can lead in half of the cases to the release of ^1^O_2_ with formation of prop-1-en-1-ol and, otherwise, to a reformation of the original 2-HPP and, in rare occurrences, to the formation of a dioxetane ring. The photodissociation of OH appears to gain importance with the energy of the photon absorbed, while the dissociation of OOH is only a minor product. A few trajectories could suffer an intersystem crossing process as they remain in S_1_ for an extended period of time without any reactions or deactivation to the ground electronic state. The following Section provides more details about the mechanisms of formation for the photoproducts by analyzing exemplary TSH trajectories.

#### 3.3.1 Exemplary TSH Trajectories Illustrating the Photochemistry of 2-HPP

This Section illustrates the formation of 2-HPP photoproducts by presenting exemplary trajectories from the swarm leading to the overall results shown in Figure 6. Trajectories will be analyzed by plotting the time trace of their electronic energies as well as key distances between atoms.

**Figure 7:**
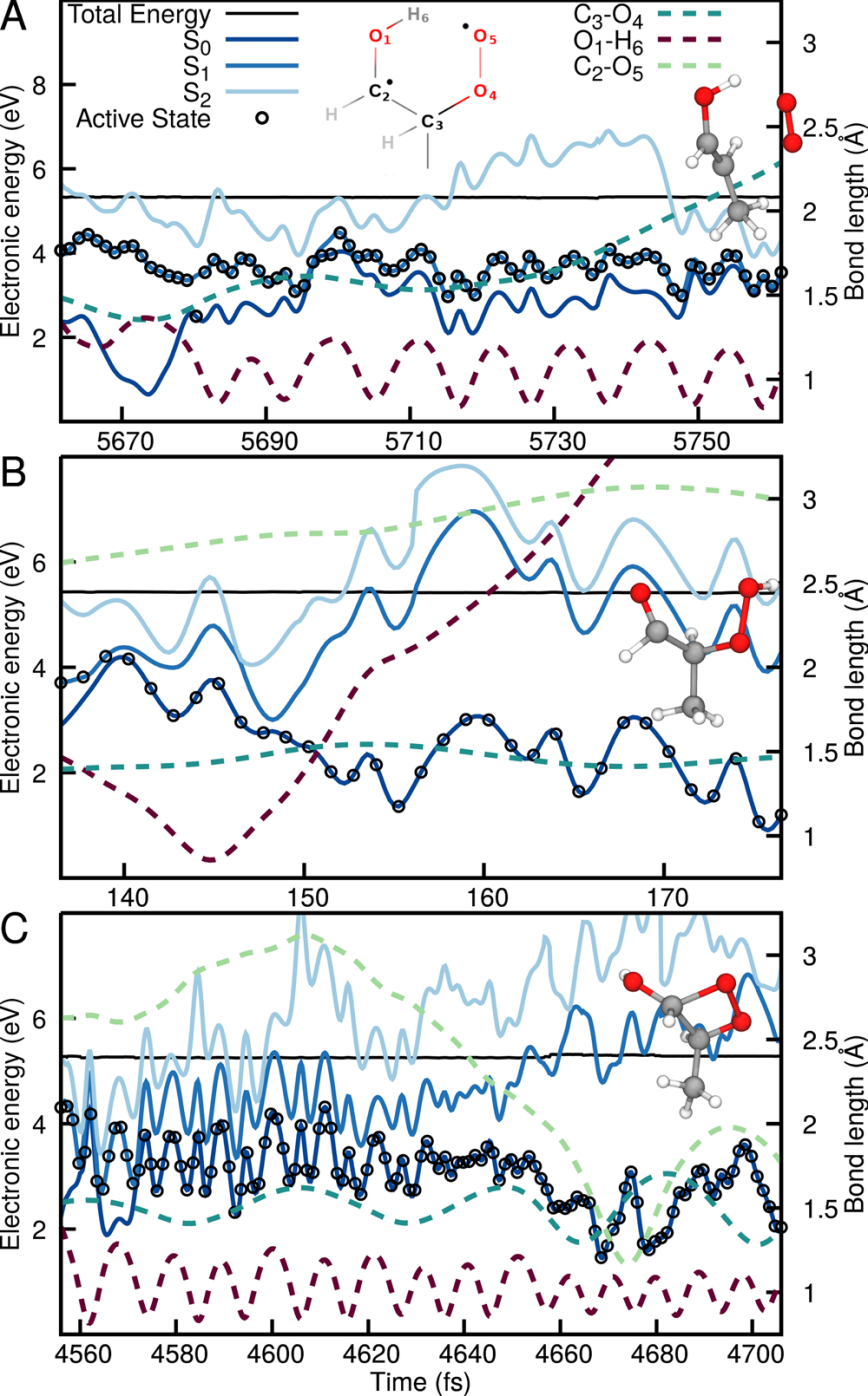
Exemplary trajectories for the proton-coupled electron transfer mechanism leading to (A) ^1^O_2_ release, (B) return to the FC region to reform 2-HPP (unreactive trajectory), and (C) the rare formation of a dioxetane ring. The energy traces (XMS(3)-CASPT2(12/9)/cc-pVDZ) highlight the three lowest electronic states, S_0_ (dark blue), S_1_ (blue), and S_2_ (light blue), with the driving state in the TSH dynamics highlighted by a black empty circle (plotted each five time steps). The total classical energy is given with a black solid line. The following distances between atoms are also plotted with dashed lines: O_1_–H_6_ (purple), C_3_–O_4_ (dark green), and C_2_=O_5_ (light green, not shown in panel A for clarity), with the atom numbering indicated in panel A for a structure following the proton-coupled electron transfer. Molecular structures illustrating the photoproducts formed are included as insets.

Let us start by considering the different outcomes following the proton-coupled electron transfer in S_1_. As discussed in Section 3.1, an adequate theoretical description of these photochemical channels requires the use of XMS-CASPT2, as they involve the visit of nonadiabatic coupling regions between S_1_ and S_0_ and electronic states with a biradical character. The first possible photoproduct of the proton-coupled electron transfer mechanism is the release of ^1^O_2_ and formation of prop-1-en-1-ol (panel A in Figure 7). The segment of the trajectory presented starts with 2-HPP in S_1_ and the proton-coupled electron transfer taking place, as identified by the shortening of the O_1_–H_6_ bond (purple dashed line in Figure 7A). The trajectory approached a region of strong nonadiabaticity with S_0_ at ∼5695 fs, transferring to the ground electronic state with a biradical character before hopping back to S_1_. The proton H_6_ remains attached to O_1_, and after 20 fs of dynamics the C_3_–O_4_ bond (dark green dashed line) begins to elongate, eventually breaking and leading to the dissociation of ^1^O_2_ after 5730 fs (see molecular inset for the structure of the photoproduct). As expected from the LIIC pathways discussed earlier (see Figure 2), the dissociation of ^1^O_2_ leads to a closing of the S_0_/S_1_ energy gap. This process is, however, not the only one possible following the proton-coupled electron transfer mechanism, as illustrated in Figure 7B. In this particular case, the trajectory reaches the S_1_/S_0_ nonadiabatic region and jumps to S_0_, where the proton migrates to O_1_ (purple dashed line in Figure 7B). At 145 fs, the proton transfers back to the original O_5_ atom reforming the hydroperoxide moiety in S_0_—no dissociation occurs (see C_3_–O_4_ bond), the electronic character of the running state changes from biradical to closed-shell, and 2-HPP is reformed in the FC region. This trajectory exemplifies an overall unreactive trajectory, despite experiencing a proton-coupled electron transfer in S_1_ before getting back to a closed shell ground-state, that is, returning toward the FC region. Finally, we highlight a rare example of the formation of a dioxetane group following the proton-coupled electron transfer (Figure 7C). In this particular case, the dynamics involving a nonadiabatic transition between S_1_ and S_0_ is similar to what is observed in Figure 7A, with the difference that the trajectory, after briefly oscillating between S_1_ and S_0_ (4560 to 4575 fs in Figure 7C), stabilizes in S_0_ after 4575 fs of dynamics. After 40 fs of dynamics in S_0_, the distance between C_2_ and O_5_ (light green dashed line in Figure 7C) where the two unpaired electrons are located decreases rapidly to form a dioxetane moiety (see molecular structure in the inset of Figure 7C). The formation of the dioxetane, taking place in the ground electronic state, is correlated with an increased separation in energy between S_0_ and S_1_/S_2_, highlighting the stabilization of this photoproduct.

**Figure 8:**
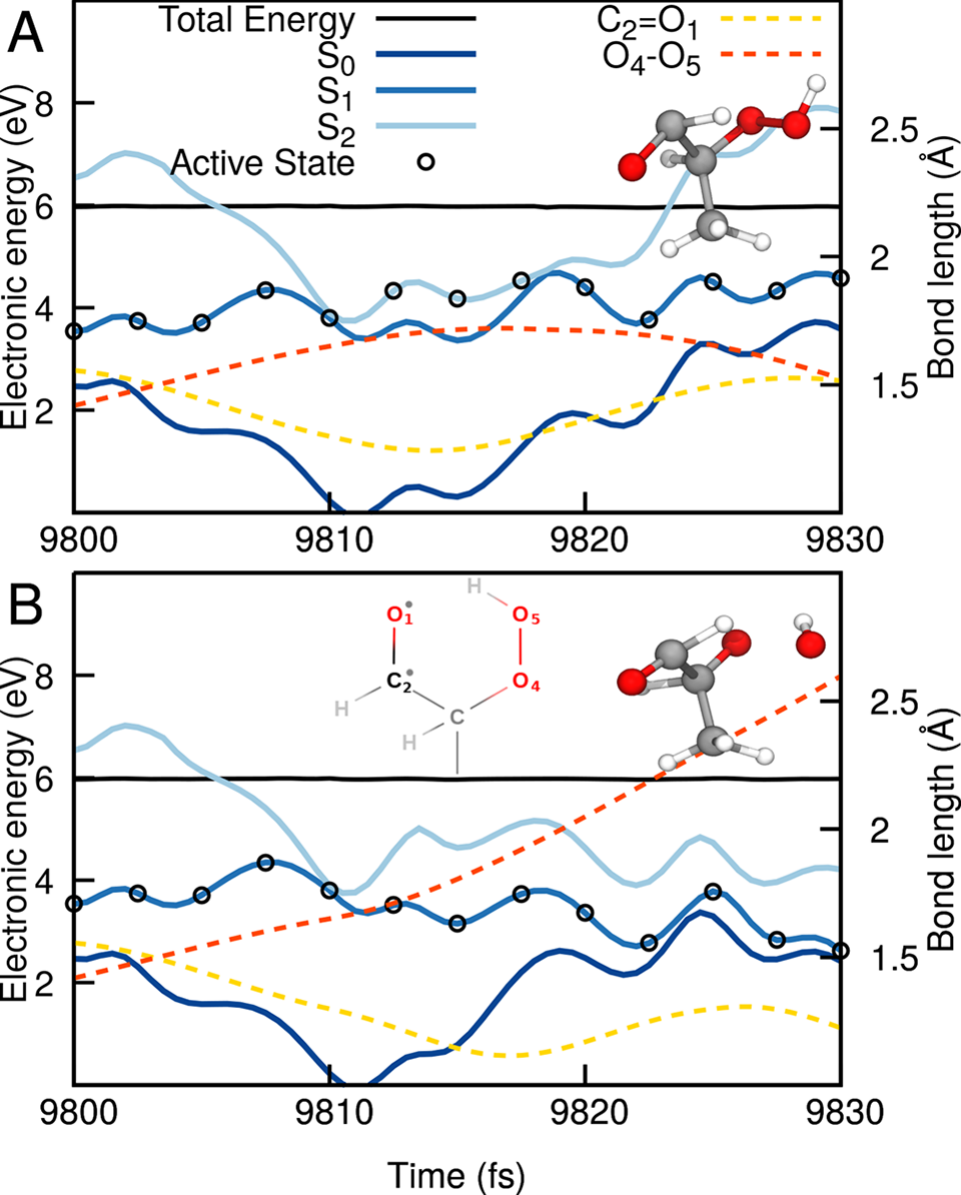
Exemplary trajectories illustrating the diabatic trapping mechanism hampering the photodissociation of OH. (A) TSH trajectory exhibiting diabatic trapping. (B) The very same trajectory, but this time artificially constrained to remain on the S_1_ electronic state. The energy traces (SCS-ADC(2)/def2-SVP) highlight the three lowest electronic states, S_0_ (dark blue), S_1_ (blue), S_2_ (light blue), with the driving state in the TSH dynamics highlighted by a black empty circle (plotted each five time steps). The total classical energy is given with a black solid line. The O_4_–O_5_ bond of the hydroperoxide moiety is indicated by a red dashed line, while the carbonyl C_2_=O_1_ bond is given by a yellow dashed line. The atom numbering is provided in panel B. Molecular structures illustrating the photoproducts formed are included as insets.

We move to the analysis of the TSH trajectories leading to an OH photodissociation. As alluded to in Section 3.1, the dissociation of OH could be obtained by photoexciting directly 2-HPP into its S_2_ electronic states, which shows an *n*′(OO) →σ*(OO) character in the FC region. This electronic state is, however, higher in energy, and it is unlikely—even if not impossible—to be reached in the actinic region (see Figure 5 and discussion in Section 3.2). The LIIC pathway presented in Figure 3 suggests that the OH photodissociation could also take place from S_1_, given that 2-HPP visits a region where the electronic character of this electronic state changes from *n*(O) → π*(CO) to *n*′(OO) → σ*(OO). Considering that the two electronic-state characters discussed here are located on different chromophores (carbonyl for *n*(O) → π*(CO) and hydroperoxide for *n*′(OO) → σ*(OO)), one expects that their diabatic coupling should be rather weak. This weak coupling is already visible from the unavoided crossing of the adiabatic states S_1_ and S_2_ in Figure 3 and reminiscent to a similar behavior observed in the photochemistry of C6-HPALD.^64^ Such a weak diabatic coupling implies that the process of switching between one electronic character to the other is somehow hampered and will be revealed in the adiabatic representation by a very localized seam of intersection between S_1_ and S_2_. When 2-HPP will approach this region of configuration space, the seam of intersection will behave as a trap for the electronic character of the molecule by transferring in a highly efficient manner 2-HPP from S_1_ to S_2_—meaning that the molecule preserves its *n*(O) → π*(CO) character in a diabatic picture. This process has been dubbed “diabatic trapping”^66^ or “upfunnelling”^67^ and, in the case of weakly coupled multichromophoric molecules like 2-HPP or C6-HPALD, protects them from OH photodissociation. The only way for the molecule to release OH from the S_1_ state is to approach the seam in such a way that it remains on S_1_ while the electronic character switches from *n*(O) → π*(CO) to *n*′(OO) → σ*(OO). With this definition in mind, we can now discuss the behavior of a trajectory exhibiting diabatic trapping (Figure 8A). This portion of configuration space is adequately described by SCS-ADC(2)—we confirmed that by running XMS-CASPT2 calculations on top of an SCS-ADC(2) trajectory (see Figure S7 in the Supporting Information). The segment of the trajectory discussed here starts at 9800 fs in S_1_. One can observe that the trajectory reaches the intersection seam with S_2_—a process correlated with an extension of the O_4_–O_5_ bond of the hydroperoxide moiety (red dashed line in Figure 8A). The trajectory hops to S_2_ and returns back to S_1_ within less than 10 fs, but the trajectory on S_1_ now sees its O_4_–O_5_ bond contracting. The diabatic transfer to S_2_ did not result in any OH dissociation. Let us artificially modify this trajectory by enforcing that it has to remain in S_1_ (Figure 8B)—all other parameters are strictly identical to those used to propagate the trajectory presented in panel (A). Again, the trajectory approaches the intersection seam, but as the trajectory is now forced to remain in S_1_, the O_4_–O_5_ bond of the hydroperoxide moiety (red dashed line in Figure 8B) now carries on its extension. As S_1_ exhibits a character change to *n*′(OO) → σ*(OO), molecule dissociates to form OH. The change of character from *n*(O) → π*(CO) to *n*′(OO) → σ*(OO) is further confirmed by the contraction of the carbonyl C_2_=O_1_ bond when the molecule leaves a *n*(O) → π*(CO) character. This comparison of trajectories makes it clear that the transfer to S_2_ preserves the *n*(O) → π*(CO) character of 2-HPP and somehow protects it from suffering an OH dissociation.

**Figure 9:**
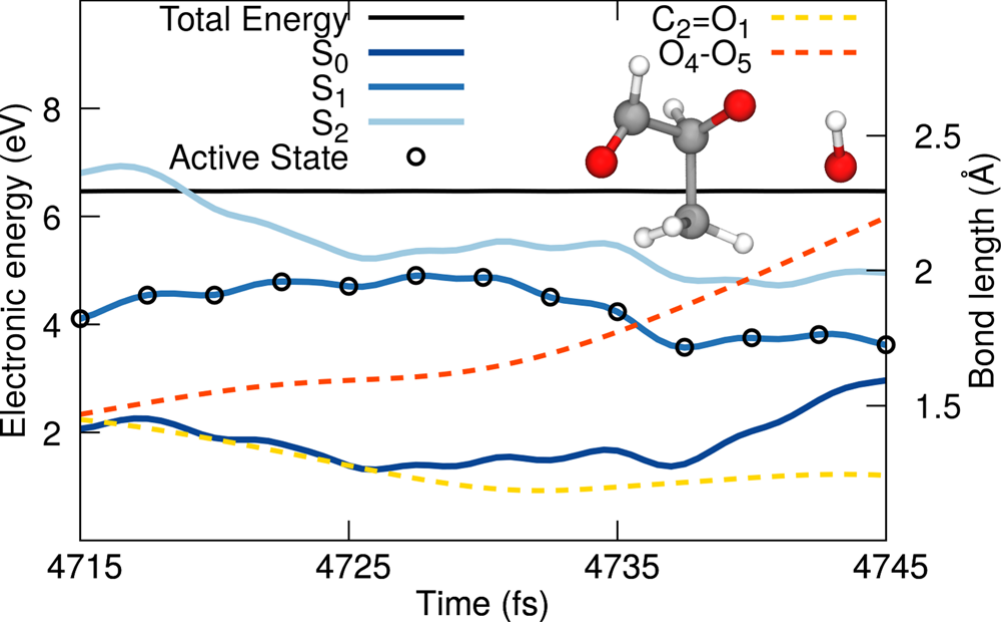
Exemplary trajectories illustrating the photodissociation of OH. The energy traces (SCS-ADC(2)/def2-SVP) highlight the three lowest electronic states, S_0_ (dark blue), S_1_ (blue), S_2_ (light blue), with the driving state in the TSH dynamics highlighted by a black empty circle (plotted each five time steps). The total classical energy is given with a black solid line. The O_4_–O_5_ bond of the hydroperoxide moiety is indicated by a red dashed line, while the carbonyl C_2_=O_1_ bond is given by a yellow dashed line. The atom numbering is the same as that employed in Figure 8. The inset shows a molecular structure illustrating the photoproduct formed.

The diabatic trapping illustrated above prevents an efficient OH photodissociation process, but a photoexcited 2-HPP in S_1_ will often visit the S_1_/S_2_ seam and, while being trapped occasionally (one diabatic trapping event in average per trajectory), may escape toward the region of configuration space where S_1_ acquires an *n*′(OO) → σ*(OO) character and dissociates OH (explaining the OH quantum yields observed in Figure 6). Figure 9 shows an example of a TSH trajectory suffering an OH dissociation. The trajectory avoids in this case the intersection seam; that is, the energy gap between S_2_ and S_1_ is not close to zero, allowing it to switch adiabatically character and release OH (red dashed line in Figure 9) with a simultaneous contraction of the carbonyl C_2_=O_1_ bond. The increase of OH quantum yield with excitation energies points toward a more efficient process at avoiding the intersection seam, perhaps due to 2-HPP having higher internal energy on S_1_.

### 3.4 Intersystem Crossing Processes

**Figure 10:**
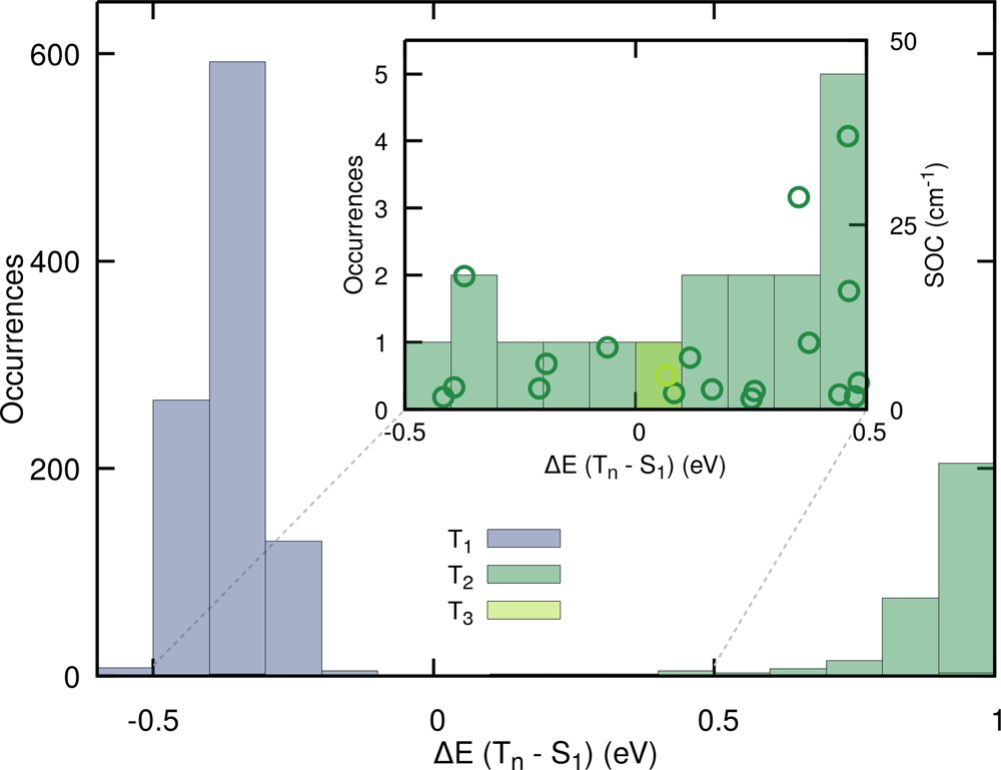
Analysis of the energy gaps between an unreactive 50 ps-long TSH trajectory evolving in S_1_ (SCS-ADC(2)/def2-SVP) and low-lying triplet states. The histograms indicate the energy gap (calculated with SCS-ADC(2)/def2-SVP) between the running S_1_ electronic state and the lowest three triplet electronic states sampled each 50 fs along the 50 ps-long trajectory. The inset provides an enlarged view of the low-energy gaps (between −0.5 and 0.5 eV), highlighting the interaction between S_1_ and the triplet states T_2_ and T_3_. For each occurrence, the absolute value of the spin–orbit coupling between S_1_ and the respective triplet state was calculated at the SA(3S,3T)-CASSCF(12/9)/cc-pVDZ level of theory (empty circles).

The calculations presented up to this point did not include the possibility for intersystem crossing processes, that is, the transfer of photoexcited 2-HPP from a singlet to a triplet state. Intersystem crossing is mediated by spin–orbit coupling, which in turn is sensitive to the electronic character of the electronic states considered. If one calculates the spin–orbit coupling matrix element between a singlet and a triplet exhibiting the same electronic character, the resulting magnitude of spin–orbit coupling will be small as a result of the El-Sayed rule—a change of orbital type between the singlet and the triplet states is required to compensate for the change in spin angular momentum. The T_1_ electronic state of 2-HPP has the same electronic character as S_1_ in the vicinity of the FC region, implying that their spin–orbit coupling is weak—for all the sampled occurrences, the mean value for the spin–orbit coupling magnitude between S_1_ and T_1_ is 1.8 cm^–1^, with a maximum value of 4.9 cm^–1^ (see Figure S9 in the Supporting Information). As our TSH trajectories only account for internal conversion, we analyzed the possible influence of intersystem crossing processes by postprocessing our long trajectories evolving in S_1_ but remaining unreactive. For a 50 ps-long S_1_ trajectory, we calculated each 50 fs the electronic-energy difference between S_1_ and the low-lying triplet states (T_1_, T_2_, and T_3_) at the SCS-ADC(2)/def2-SVP level of theory and produced a histogram of these energy gaps (Figure 10). A first important observation is that crossings between S_1_ and a triplet state are rather infrequent and the low-lying triplet states remain rather far from S_1_ during this 50 ps-long trajectory. Nevertheless, the calculations presented here are in a spin-diabatic representation, meaning that, in this picture, one does not need to have crossings between S_1_ and a triplet state for an intersystem crossing to take place. To account for this fact, we enlarged the energy-gap window between −0.5 and 0.5 eV and, for each occurrence of a crossing between S_1_ and T_2_ or T_3_ (to account for the El-Sayed rule), we calculated the magnitude of the corresponding spin–orbit matrix element with SA(3S,3T)-CASSCF(12/9)/cc-pVDZ (circles in the inset of Figure 10). The magnitudes of spin–orbit coupling calculated are all rather small yet sizable. We further mimicked the effect of an intersystem crossing by restarting, for each occurrence, the trajectory in the corresponding triplet state (mostly in T_2_). For 17 out of 18 trajectories launched, we observed an almost immediate OH release, as a result of the dissociative character of T_2_. Hence, if the time scale of the molecule in S_1_ is long enough to allow for intersystem crossing processes, T_2_ appears to be the most likely receiving triplet state, leading to an immediate photodissociation of OH. If the molecule can reach T_1_ directly from S_1_, other decay mechanisms may be expected, as discussed in ref (23).

### 3.5 Limitations of the Theoretical Protocol

We discussed in the previous Sections how the photoabsorption cross-section of 2-HPP can be predicted using the nuclear ensemble approach and how nonadiabatic and ground-state dynamics simulations can be used to approximate quantum yields for photoproducts. As already alluded to in numerous parts of this work, the theoretical strategies used herein have a series of limitations when it comes to the simulation of atmospheric VOCs that we would like to further stress in this Section.

First of all, our work exemplifies the challenges related to the use of an adequate method for the electronic structure. Methods like ADC(2) or even LR-TDDFT can provide a reasonable description of electronic transitions for organic molecules. Care has to be taken though when looking at atmospheric VOCs, as some molecules may possess excited electronic states with a significant doubly excited-state character. LR-TDDFT cannot describe such electronic states, and ADC(2) can only account for them at zeroth order. The same is true for molecules with a zwitterionic character like Criegee’s intermediates, which are notorious for their complex electronic structure.^28^ Higher-level methods based on the CC formalism like CC3 or CC4 would provide more accurate transition energies but at a much higher computational cost.^68−71^ More importantly, an electronic structure method can provide a proper description of the excited electronic states of interest at the ground-state optimized geometry but may fail miserably as soon as the molecule leaves this region. Such inhomogeneity of the quality of potential energy surfaces has been exemplified greatly in the past with the charge-transfer issue in LR-TDDFT (see, e.g., the case of DMABN in ref (72)) or with the shortcomings of ADC(2) when describing carbonyl-containing molecules.^46^ In this work, one of the challenges for the electronic structure was to describe in a balanced way electronic states with different characters—sometimes an issue for SA-CASSCF—and to account for the biradical nature of some photoproducts formed. XMS-CASPT2 appeared to be a good compromise in these regions. However, the multichromophoric nature of 2-HPP makes the active space required for XMS-CASPT2 rather large and therefore computationally expensive. The computational cost explains why XMS-CASPT2 could not be used as an electronic structure method for our TSH dynamics with a largely prolonged time scale. As mentioned in the text, some of our simulations were run for up to 100 ps, which is a considerable computational effort for excited-state dynamics and would simply not be possible with XMS-CASPT2—in particular, if one considers that we ran here a total of 246 trajectories to ensure a modest swarm of TSH trajectories. While we have validated the protocol of switching from SCS-ADC(2) to XMS-CASPT2, this strategy is far from ideal. Recent developments related to XMS-CASPT2 open a new perspective for future applications of this method.^73^ However, the photochemistry of atmospheric VOCs can also be challenging for XMS-CASPT2. For example, dissociative pathways may imply that more electronic states become important at some point in the dynamics. Gaining more flexibility in the number of electronic states considered in the state-averaging and multistate process would be highly beneficial, as proposed by recent developments in dynamically weighted SA-CASSCF.^74^

Obtaining absolute photoabsorption cross sections implies that one also accounts for the quantum nature of the nuclei in the ground electronic states. The NEA employed in this work offers an efficient way to approximate the photoabsorption cross-section. The underlying sampling of the ground-state probability density for the lowest vibrational level uses a Wigner distribution within an harmonic approximation. The flexibility of some atmospheric VOCs means that a harmonic Wigner distribution may not always lead to a satisfactory representation of the ground-state probability density, and other alternatives like ab initio molecular dynamics with a quantum thermostat are sometimes required—such effects appear negligible for 2-HPP.^30^

Last but not least, the excited-state dynamics simulations have some implicit limitations and rely on significant approximations. A first limitation may come from the use of the mixed quantum/classical method TSH. This method treats the nuclei in a classical way and therefore does not allow for tunneling processes. The method also may suffer from its approximation when multiple crossings between the same pair of excited electronic states take place—like the diabatic trapping observed here—but for molecular systems using a decoherence correction (as done here) is usually sufficient to fix this potential issue.^75^ The simulations conducted here do not account for intersystem crossing processes. Different strategies have been proposed to account for both internal conversion and intersystem crossing processes in nonadiabatic molecular dynamics,^76−78^ but the challenge is often related to the time scale of these events as well as the added challenge for the electronic-structure method to describe both triplets and singlets. We note that TSH extended to describe intersystem crossing events was successfully used to investigate mercury-based compounds in the atmosphere.^27^ As discussed earlier, the time scale of the TSH trajectories for 2-HPP in this work was up to 100 ps. However, the use of current nonadiabatic molecular dynamics strategies for such long-time-scale simulations raises a series of questions, as recently illustrated in ref (79). Including the role of spin–orbit coupling and possibly collisions for long time scale processes could be performed with reduced-dimensionality models like the energy-grained master equation (EGME) extended to nonadiabatic processes, even if the construction of the model is often informed by all-atom dynamics.^64^ Finally, the excited-state dynamics presented were directly initiated in the excited state by selecting initial conditions from the photoabsorption cross-section within a series of energy windows. While this offers a first approximation to wavelength-dependent processes like quantum yields, more involved strategies would be required to simulate the time scales of photoinduced processes for an atmospheric molecule under incoherent sunlight irradiation.^80−82^

### 3.6 Photolysis Rate Constants

Armed with a calculated photoabsorption cross-section (Figure 5) and the different wavelength-dependent photolysis quantum yields (Figure 6), we can attempt to predict in silico the photolysis rate constants for the main photoproducts of 2-HPP. We adopt here an actinic flux *F*(λ) for a 30° solar zenith angle and 300 DU ozone, obtained from the Tropospheric Ultraviolet and Visible (TUV 5.4) radiation model,^83^ and we integrate eq 1 in an interval from 280 to 360 nm. Note that the wavelength-dependent quantum yields are estimated for the dominant 2-HPP conformer (1a). Nevertheless, the quantum yields for the conformer 1c that does not exhibit H-bond are reasonably similar (see Figure S6, indicating that the excited-state dynamics is sufficiently long to alleviate the memory of the initial conformer structure). *J* values for the two main photolysis channels, namely, the formation of ^1^O_2_ and OH, amount to *J*_^1^O_2__ = 2.9 × 10^–5^ s^–1^ and *J*_OH_ = 3.2 × 10^–5^ s^–1^, while the cumulative *J* for all photolysis channels is calculated to be 6.7 × 10^–5^ s^–1^. In comparison, Liu et al.^23^ determine a *J* for the 1,5-H shift process (followed by O_2_ release) to be 6.8 × 10^–4^ s^–1^,^a^ using the same actinic flux as above and a unity quantum yield. The latter *J* value is more than 25 times larger than our best estimate for the rate of the O_2_ release, while it is still 10 times larger than our total *J* (including all photolysis processes). Using the scaled photoabsorption cross-section of Liu et al. (scaled composite spectrum in Figure 5) and our calculated ϕ_^1^O_2__(λ) for the release of ^1^O_2_, we obtain a *J* value of 1.8 × 10^–4^ s^–1^. Alternatively, combining our calculated σ(λ) (Figure 5) with ϕ = 1 (used by Liu et al.) gives a *J* value of 1.1 × 10^–4^ s^–1^. Therefore, it is clear that both the reduced ϕ(λ) and σ(λ) from the present work, in comparison with ref (23), significantly affect the *J* estimates. In the context of atmospheric chemistry, our new estimate for the cumulative photolysis rate constant is smaller than the rate constant for the reaction of 2-HPP with OH radicals (estimated to be 1.3 × 10^–4^ s^–1^ based on the MCM model^23^), meaning that photolysis may not be a dominating pathway for the removal of 2-HPP. Comparable data for other important α-hydroperoxycarbonyls and their impact on atmospheric species concentration balance remain to be evaluated.

## 4 Conclusion

The theoretical determination of photolysis properties for atmospheric VOCs is a challenging yet rewarding task, as photolysis rates for many atmospherically relevant, transient VOCs are hardly available based on experiments alone. While using SARs often provides valuable insights on unknown molecules, fully in silico investigations provide a more robust and reliable way of investigating the photolysis of various VOC species. In this work, we used a large set of computational strategies to investigate the photochemistry of a complex multifunctional molecule from the family of α-hydroperoxycarbonyls. We presented a series of sensible approximations to calculate the photoabsorption cross-section and wavelength-dependent photolysis quantum yields, the key ingredients to evaluate sought-for photolysis rate constants. Following our earlier work on the photolysis of hydroperoxides,^24^ we employed the nuclear ensemble approach to calculate the photoabsorption cross-section of 2-HPP, highlighting a discrepancy with previous estimates based on SARs considerations. A combination of nonadiabatic and ground-state molecular dynamics simulations allowed us to determine wavelength-dependent quantum yields for various photoproducts formed upon photoexcitation of 2-HPP. Nonadiabatic molecular dynamics is expected to provide an unbiased and automated way to explore complex potential energy surfaces and unravel the most relevant dissociation pathways and their corresponding yields, accounting for athermal effects that may elude regular transition-state theory. Using our in silico quantum yields and photoabsorption cross-section, we estimated the rate constants of the most important photolysis channels, showing that photolysis processes may not be dominant in atmospheric condition to explain the removal of 2-HPP—reaction with OH being faster. While our own protocol is subject to limitations (many of which are identified in Section 3.5), we believe that it represents a reliable framework to explore the photochemistry of various transient VOCs and calculate their photolysis rate constants when these are unavailable experimentally.

## Supporting Information Available

Additional details about the total number of trajectories, the free energies for the 2-HPP rotamers and their interconversion, the active space orbitals, the LIIC pathway from FC point to S_1_ minimum, the quantum yields for the 1c conformer, comparison of the XMS(3)-CASPT2 dynamics with that obtained with SCS-ADC(2) and XMS(2)-CASPT2 (PDF)
Raw data for the photoabsorption cross-section and quantum yields used to compute *J*, as well as the critical geometries employed to generate the LIICs, in xyz format (ZIP)

## Footnotes

a We reproduced the *J* value reported in ref (23) (Figure 5) by using the digitized version of the photoabsorption cross-section from this work, validating that we employ a similar photon flux. We note that the same authors obtained slightly smaller *J* of 3.9 × 10^–5^ s^–1^ with the TUV code, as shown in Table 2 of ref (23).
