# Supplementary material for: A Theoretical Perspective on the Actinic Photochemistry of 2-Hydroperoxypropanal
Source: J Phys Chem A. 2022 Jul 28;126(32):5420–33. doi: 10.1021/acs.jpca.2c03783 (PMC9393889; doi:10.1021/acs.jpca.2c03783)
Supplement: Supplementary file 1 — jp2c03783_si_001.pdf [file jp2c03783_si_001.pdf]

Supporting Information: A Theoretical Perspective on the Actinic Photochemistry of 2-Hydroperoxypropanal

Emanuele Marsili[a], Antonio Prlj[a], and Basile F. E. Curchod[a*]

[a] *Centre for Computational Chemistry, School of Chemistry, University of Bristol, Bristol BS8 1TS, UK*

E-mail: basile.curchod@bristol.ac.uk

Table S1: Number of trajectory used in the analysis for the conformers 1a and 1c. Two trajectories for the conformer 1a reached 100 ps of dynamics without showing any reactivity. One trajectory for the conformer 1c was discarded due to SCS-ADC(2) electronic structure instability.

	No. of trajectories	Discarded (NRCI)	Discarded (electronic structure instability)
1a	166	34	1
1c	80	17	4

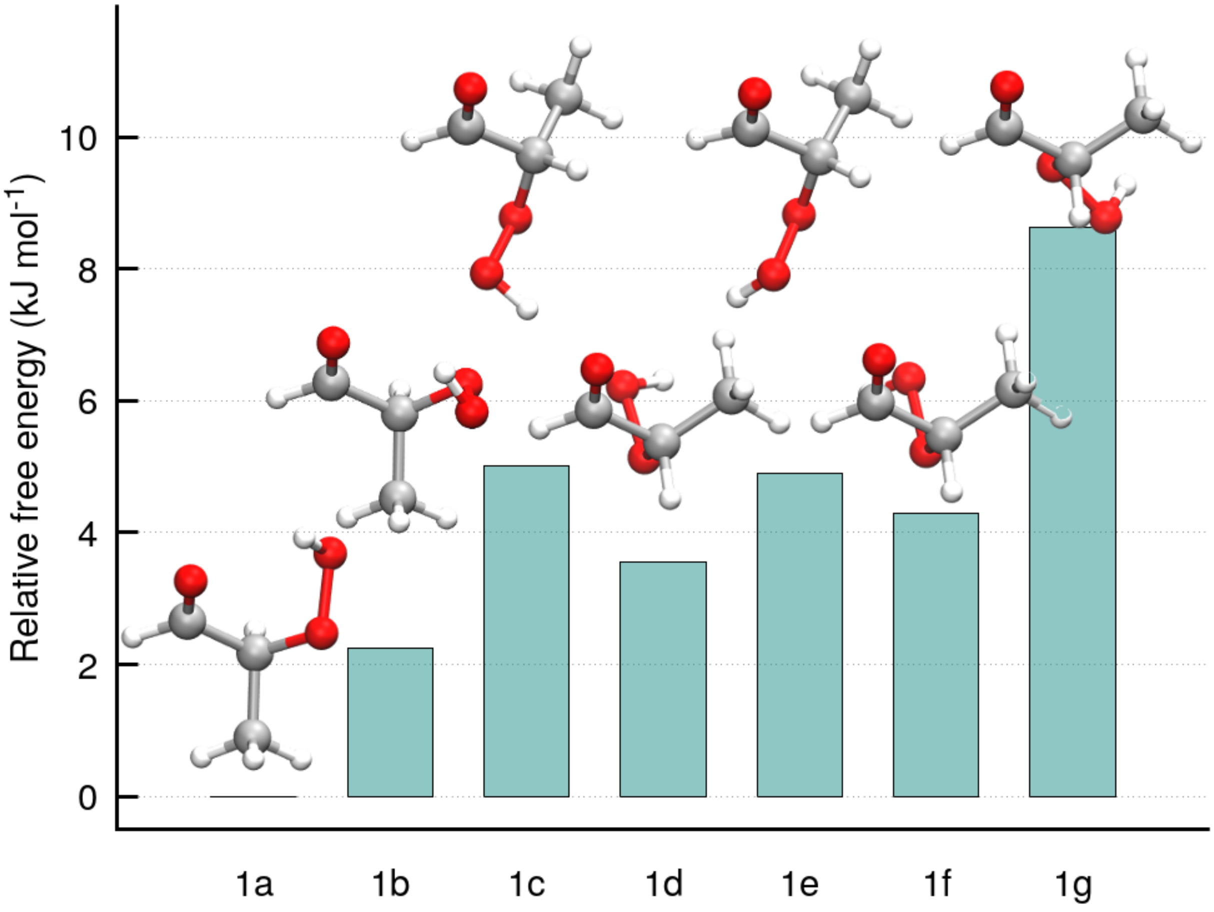

Figure S1: Geometries of the seven rotational isomers of 2-HPP and the corresponding relative free energies computed at SCS-MP2/def2-SVP.

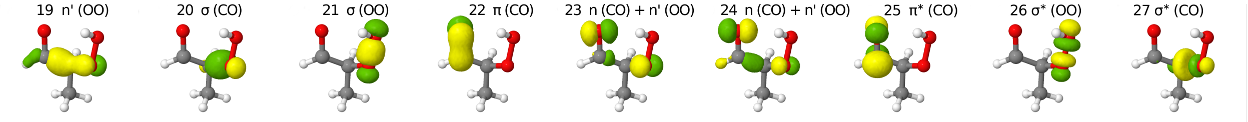

Figure S2: SA(3)-CASSCF(12/9) natural orbitals used for the XMS(3)-CASPT2(12/9)/cc-pVDZ calculations, at the S_1 minimum geometry obtained with SCS-ADC(2)/def2-SVP.

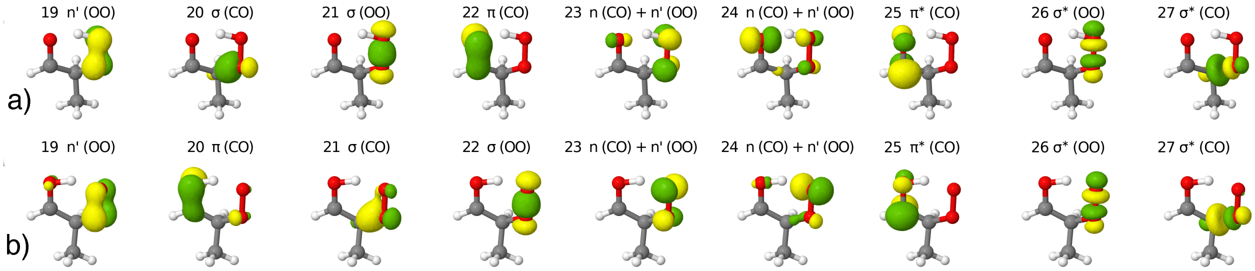

Figure S3: Evolution of the active space natural orbitals (SA(3)-CASSCF(12/9)) used for the XMS(3)-CASPT2(12/9)/cc-pVDZ calculations at (a) the S_1 TS geometry and (b) MECI geometry along the proton-coupled electron transfer pathway (see Fig. 2 in the main text).

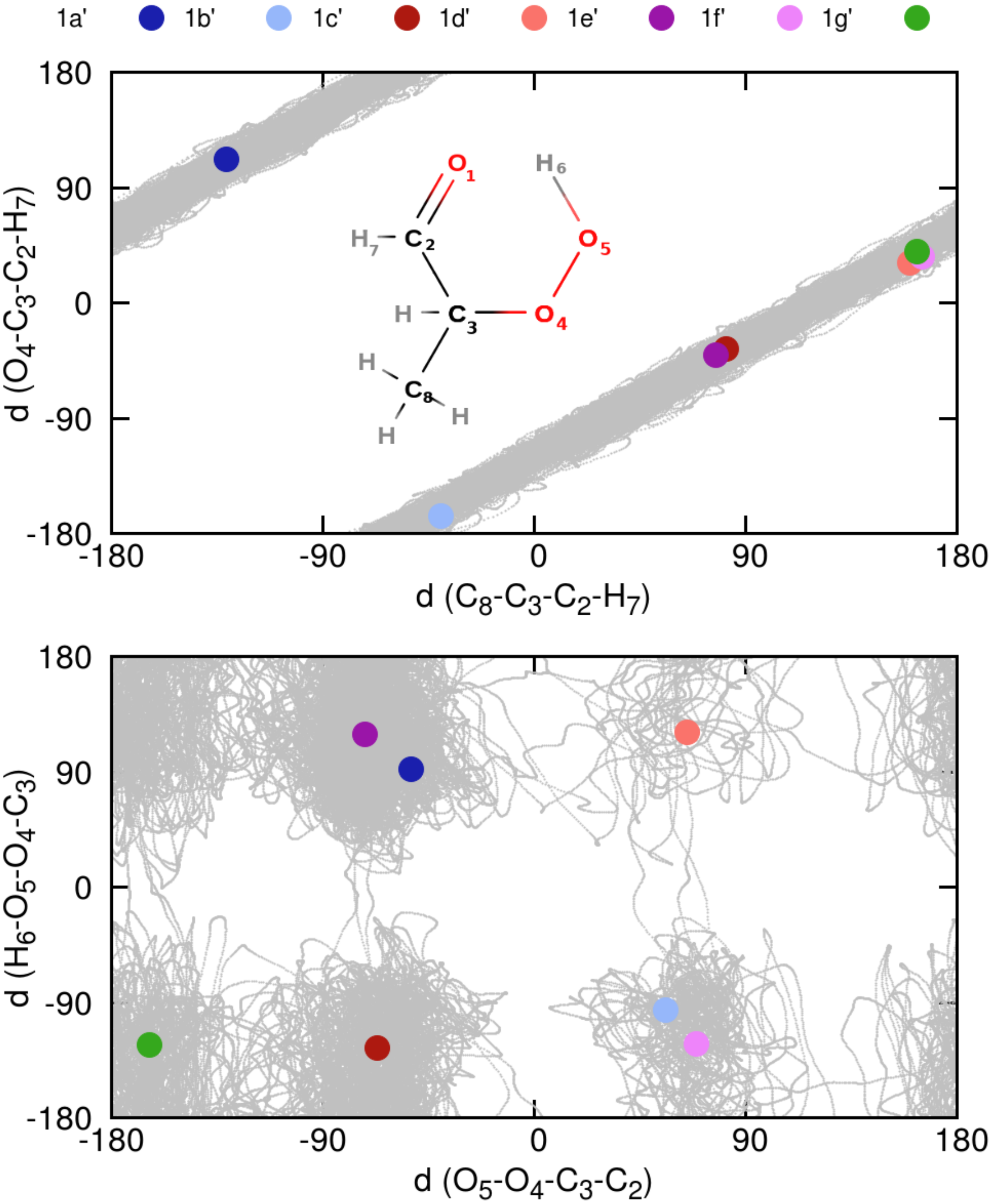

Figure S4: Interconversion between different conformers of 2-HPP during 100 ps of excited-state dynamics in S_1. This trajectory does not suffer any dissociative pathways during the 100 ps of dynamics (see Sec. 3.3 in the main text). The gray dotted line shows the S_1 trajectory projected on specific dihedral angles used to characterized the different conformers of 2-HPP. The prime notation after each conformer name indicates that we focus on the S_1 minimum of each conformer originally identified in the ground electronic states (see Fig. S1). These S_1 optimized geometries were obtained with SCS-ADC(2)/def2-SVP, and their position on the projected space labeled by a colored dot. The inset shows a structure and the atom numbering.

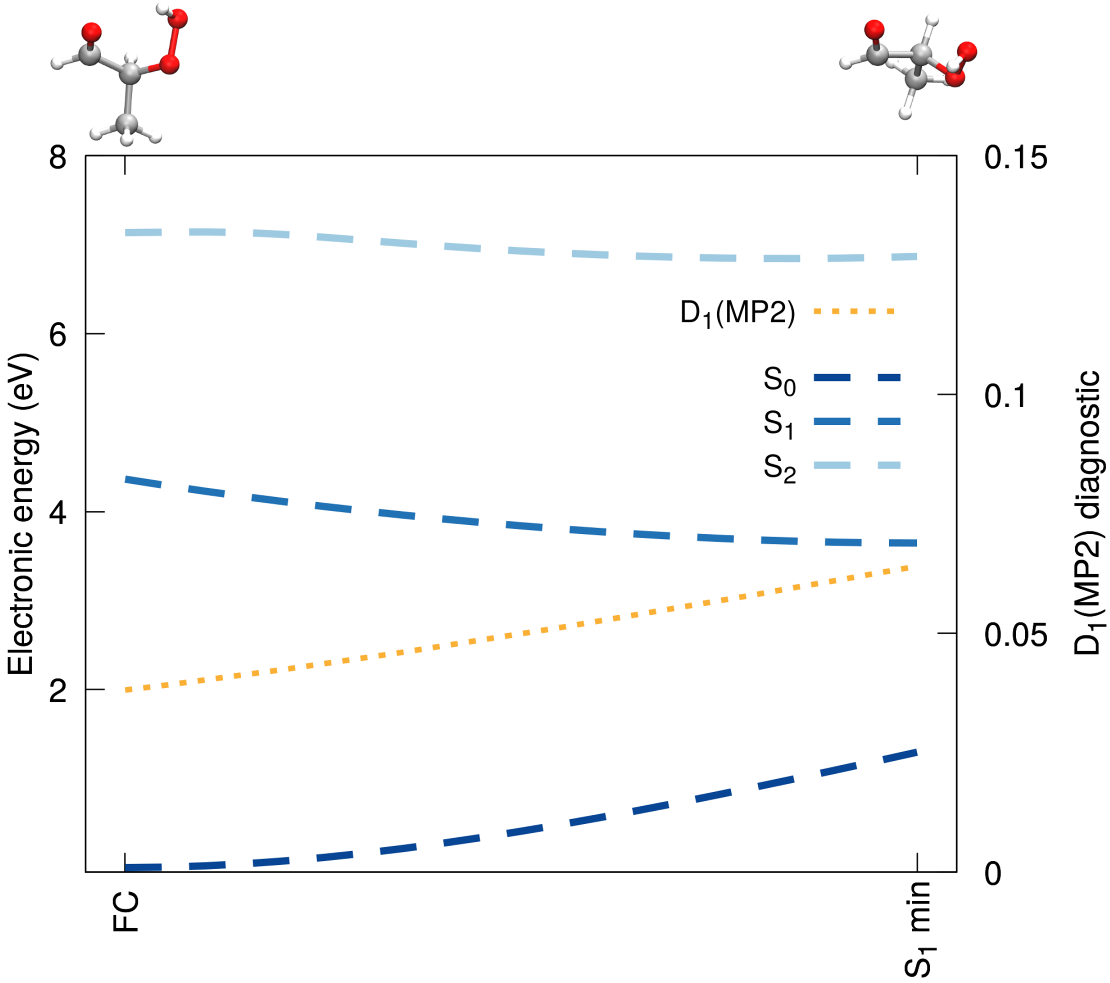

Figure S5: LIIC pathway connecting the Franck-Condon (FC) point to the S_1 minimum. Electronic energies were obtained with SCS-ADC(2)/def2-SVP (dashed lines). The upper panel shows the molecular structure corresponding to the two critical points located. The D_1 diagnostic along the pathway for the SCS-MP2 ground state is given with a dotted orange line.

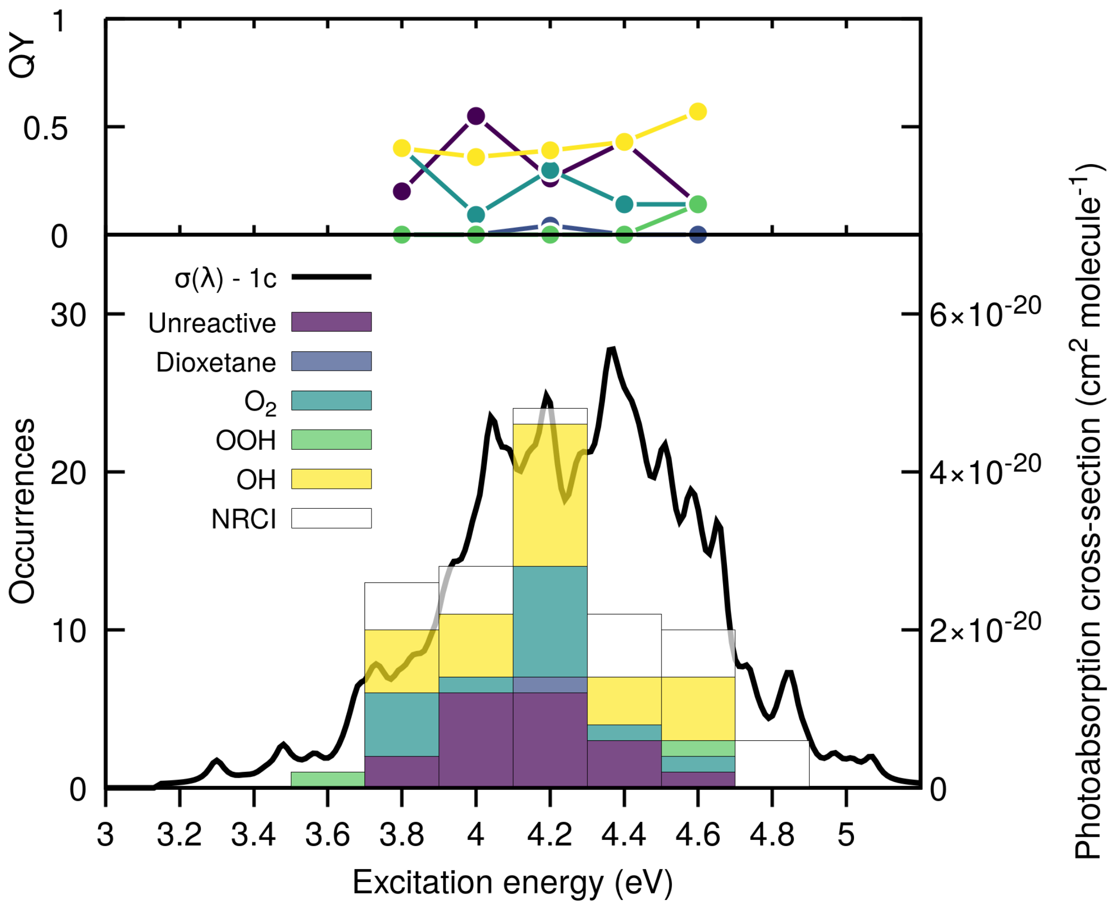

Figure S6: Wavelength-dependent photoproducts (lower panel) and quantum yield (upper panel) for the conformer 1c of 2-HPP. The photoproducts – OH and OOH dissociation, $^1O_2$ release and formation of prop-1-en-1-ol, dioxetane formation, unreactive trajectories, non-reactive conical intersection (NRCI) – were obtained by simulating the excited-state and subsequent athermal ground-state dynamics of 2-HPP (conformer 1c) with TSH/SCS-ADC(2)/def2-SVP and TSH/XMS(3)-CASPT2(12/9)/cc-pVDZ. The occurrences (number of TSH trajectories ending as one of the photoproducts defined) of each reactive pathway are overlaid with the calculated photoabsorption spectra (SCS-ADC(2)/def2-SVP) for S_1 state of the conformer 1c. The trajectories leading to a NRCI pathway were discarded from the quantum yield calculation. The wavelength-dependent quantum yields were calculated only for windows with more than seven successful trajectories

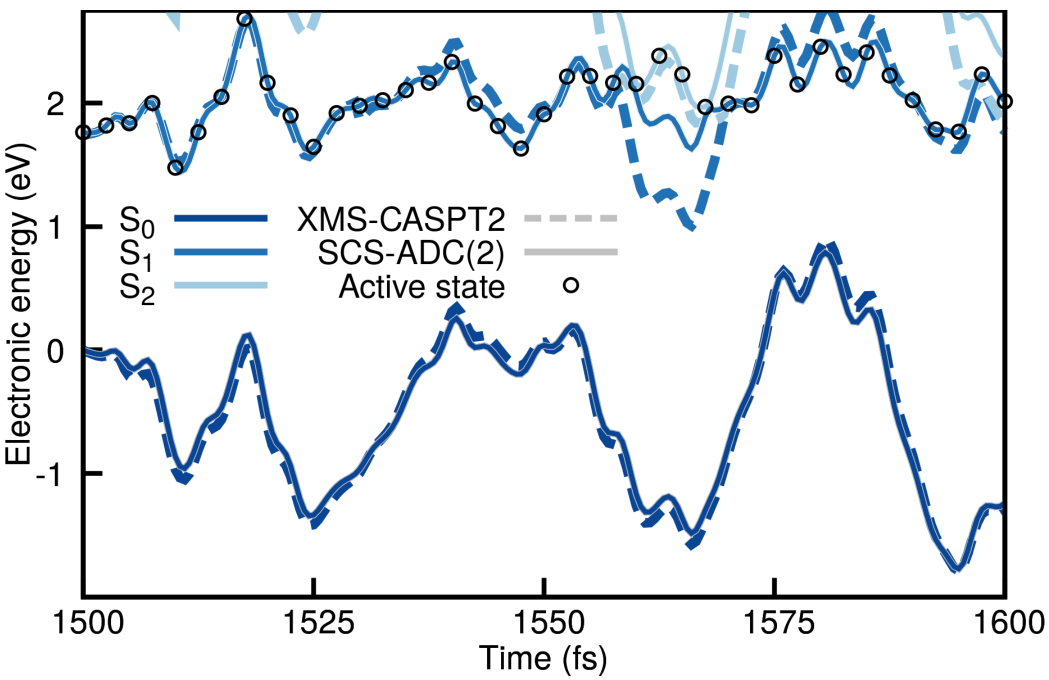

Figure S7: Comparison between the electronic energies obtained along a SCS-ADC(2)/def2-SVP TSH trajectory (solid lines) evolving on S_1 and the electronic energies recalculated on the support of these nuclear coordinates with XMS(3)-CASPT2(12/9)/cc-pVDZ (dashed line). The trajectory suffers a diabatic trapping just after $t = 1550$ fs. The three lowest electronic states, S_0 (dark blue), S_1 (blue), and S_2 (light blue) are shown, with the driving state highlighted by an empty black circle.

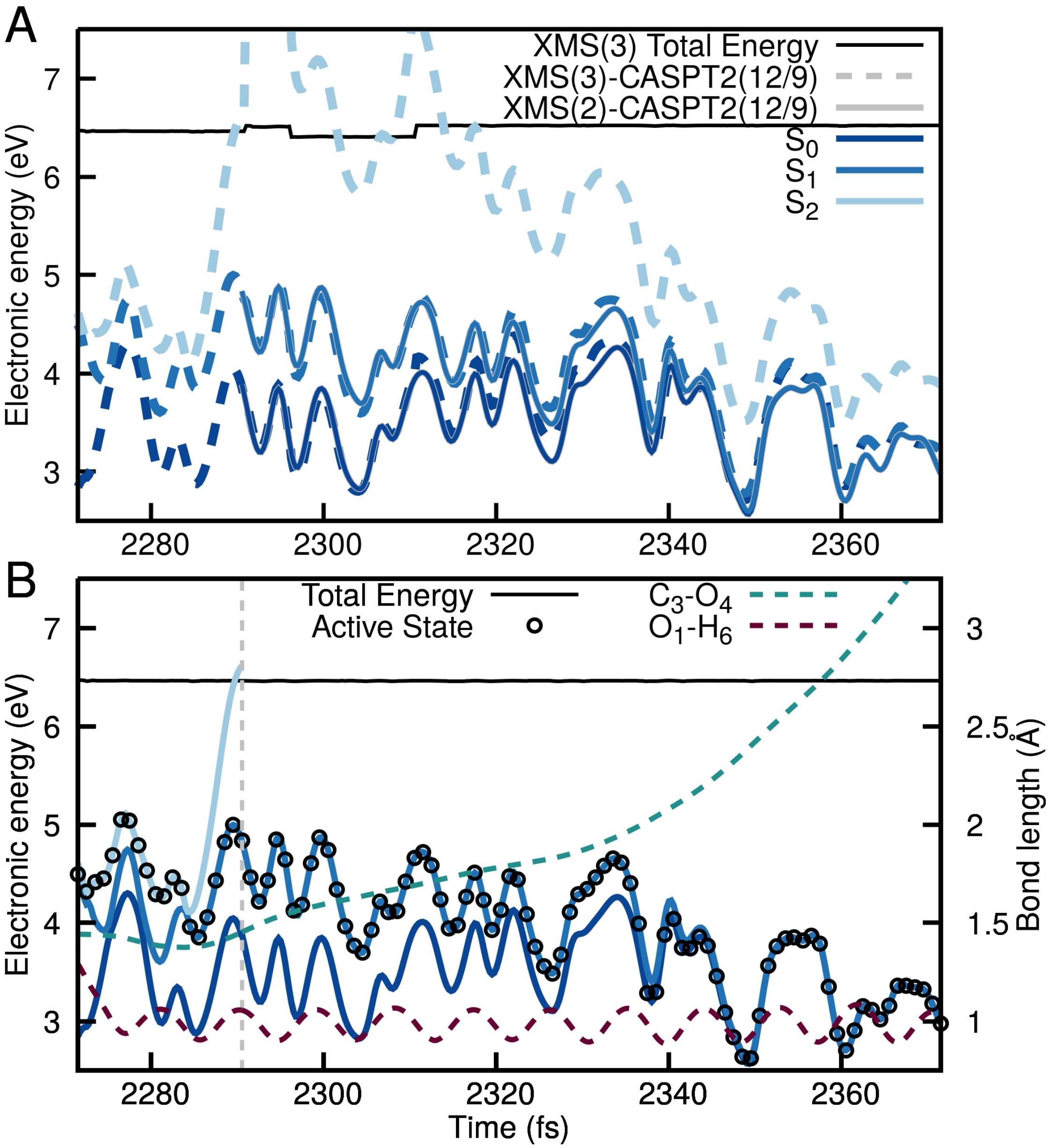

Figure S8: Exemplary TSH trajectory to illustrate how the dynamics after the proton-coupled electron transfer is switched from XMS(3)-CASPT2(12/9) to XMS(2)-CASPT2(12/9) (vertical gray dashed line). Once the trajectory left the region of nonadiabaticity between S_1 and S_2, the electronic state with a closed-shell character is destabilized (S_2 after $t = 2280$ fs) and only weakly interact with S_0 and S_1. Considering only these two states for the remaining of the dynamics stabilizes the trajectory. The S_1 electronic energy computed with XMS(2)-CASPT2(12/9) has been overlaid with the S_1 energy calculated with XMS(3)-CASPT2 at the restarting point.

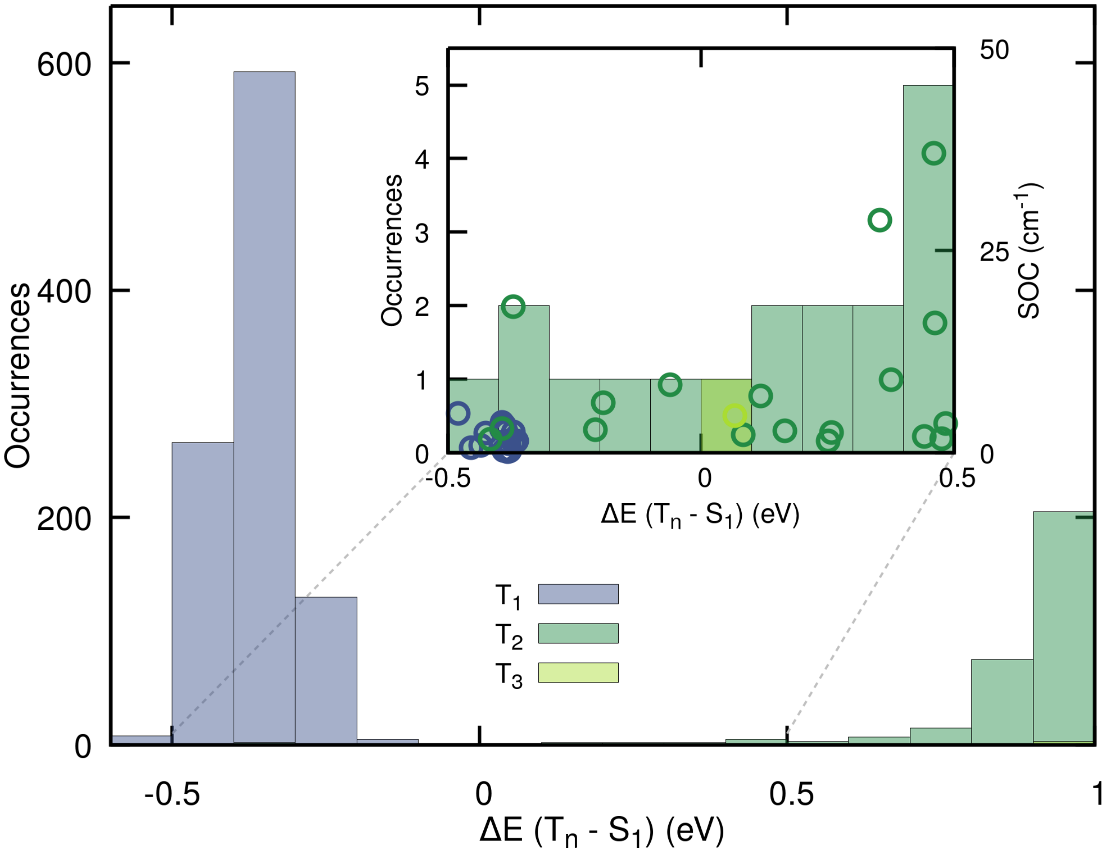

Figure S9: Same figure as Fig. 10 in the main text but with the absolute value of the spin-orbit coupling between S_1 and T_1 for each occurrence of the S_1/T_n crossings indicated with a purple empty circle. The average absolute value for the SOC between S_1 and T_1 is 1.84 cm^{-1}.
